# Fourier ptychography microscopy for digital pathology

**DOI:** 10.1111/jmi.70001

**Published:** 2025-06-24

**Authors:** Fraser Eadie, Laura Copeland, Giuseppe Di Caprio, Gail McConnell, Akhil Kallepalli

**Affiliations:** ^1^ Department of Biomedical Engineering, Wolfson Building University of Strathclyde Glasgow UK; ^2^ Strathclyde Institute of Pharmacy and Biomedical Sciences University of Strathclyde Glasgow UK; ^3^ Centre for the Cellular Microenvironment Department of Biomedical Engineering University of Strathclyde Glasgow UK; ^4^ School of Molecular Biosciences University of Glasgow Glasgow UK; ^5^ School of Physics and Astronomy University of Glasgow Glasgow UK

**Keywords:** AI, deep learning, digital pathology, fluorescence, Fourier optics, medical imaging, phase‐retrieval, polarisation

## Abstract

Fourier ptychography microscopy (FPM) has made significant progress since its invention in 2013, thanks to its adaptable nature, high resolution, and vast field‐of‐view capabilities. FPM is used in various medical applications across multiple optical wavelengths, from automated digital pathology to radiology and ultraviolet label‐free imaging. This review explores the fundamental physical and computational concepts that have driven advancements in digital pathology using FPM. A crucial part of the progress has been the development of computational algorithms, which have directly contributed to the improvements in FPM. We evaluate early‐stage algorithms like the Gerchberg–Saxton and highlight how phase‐retrieval and deep‐learning advancements have propelled FPM forward. Additionally, we discuss the impact of these algorithms on digital pathology for potential automated diagnosis, providing a comprehensive explanation of their influence on medical imaging and offering insights into future research directions.

## 1 INTRODUCTION

Most microscope users must decide between imaging a large field of view at a low resolution or a small field of view at a high resolution. This optical issue has arisen from limitations in the space‐bandwidth product (SBP). Fourier ptychography microscopy (FPM) is a recently developed physical and computational technique that successfully overcomes the diffraction limit of the optical system. In the first experimental demonstration of FPM, a standard microscopic image was compared with that from a microscope adapted with a light‐emitting diode (LED) array instead of a standard light source.^1^ This work assumed that each LED in the array acts as a point source; each LED was then sequentially switched on and off in a specific order to produce images with different illumination angles. Each angle captured a unique frequency component of the specimen's optical transfer function, resulting in a Fourier plane shift of the object's spatial frequency spectrum. When the light passed through the sample, due to the varying angles, it alternated in both the temporal‐spatial and Fourier space domains.

**FIGURE 1:**
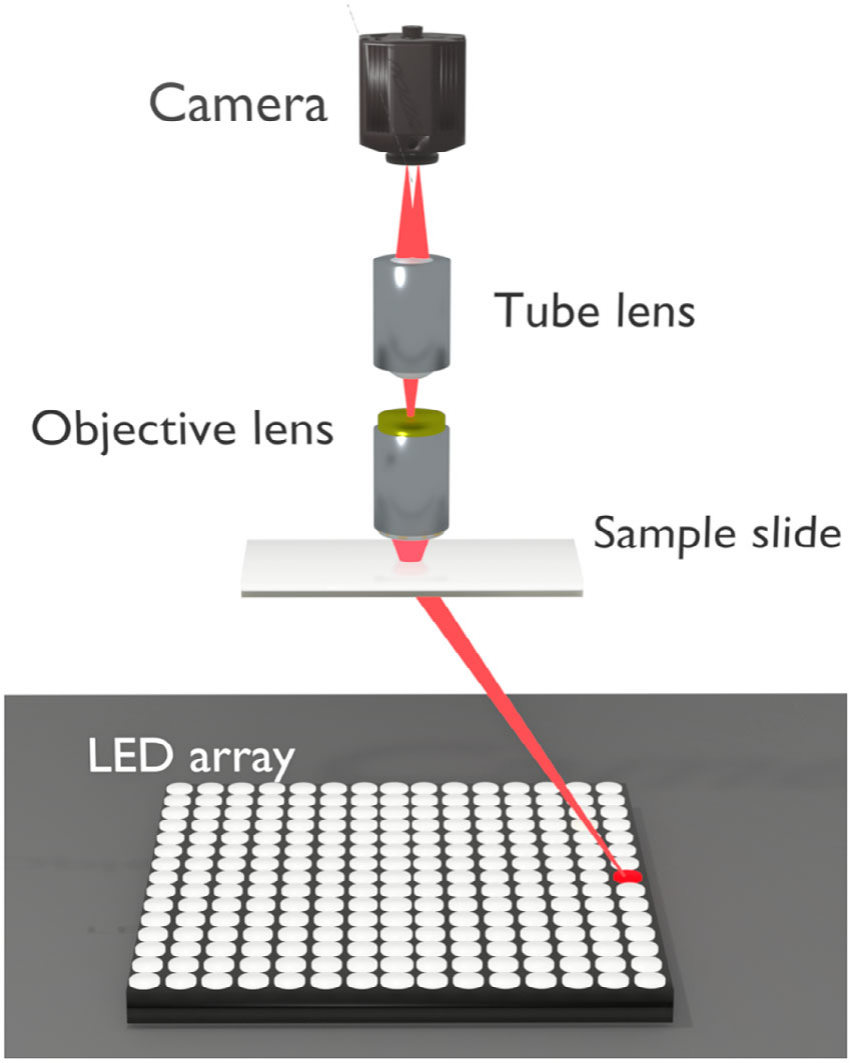
A standard Fourier ptychography microscopy setup differs from a normal microscope by replacing a single light source with an LED array capable of illuminating the sample from multiple directions. This system allows the capture of all three types of light necessary for Fourier ptychography.

In a standard FPM setup (shown in Figure 1), an LED array is used to illuminate a sample from multiple unique angles. Assuming Köhler geometry, the LED array allows the illumination patterning at the sample's Fourier plane.^2^ After interaction with the sample/object, this light is collected for imaging using a relatively low numerical aperture (NA) objective lens. By imaging a sample from varying angles, the Fourier diffraction pattern at the back focal plane of the objective lens will effectively shift to different positions in Fourier space.^3^ Illumination from the central LEDs creates brightfield images, whereas illumination from the larger angle LEDs (outside the objective) forms darkfield images. Practically, three types of images within Fourier ptychography are combined to achieve this. These are: the dark field of the image (which is observed only when the scattered light is captured), a bright field (captured when images contain scattered and unscattered light from the central LEDs), and a transitional image between the bright‐ and dark fields.^4^ Ptychography is then used to combine the acquired images in bright and dark fields. In ptychography, a detector records an extensive dataset consisting of varying inference patterns obtained as an object is moved to different positions relative to the illumination field.^5^ In Fourier ptychography, the camera acts as the detector, and the scattered light from each of the different positions of the LEDs synthetically acts as the object moving to various positions. By varying the incident angle of LEDs, a series of confined Fourier spectra overlapping with spectra from adjacent LEDs is captured by the aperture, creating a sequence of images for the sensor.^6^ It is important to note that the LEDs need to be sufficiently powerful such that the light is still detected by the camera. The phase lost within the low‐resolution image is recovered, as phase‐matching algorithms are used to frequency match the acquired images after Fourier transformation.^7^

The images obtained are analysed in the frequency domain as the low‐resolution images collectively contain overlapping frequency information which, when correctly matched, can be used to reconstruct a high‐resolution image.^8^ FPM combines fundamental concepts in Fourier optics and waves with new advancements in computing, such as phase‐retrieval algorithms and deep learning (DL). FPM uses phase‐retrieval methods to converge to a solution of a high‐resolution complex sample image by alternating between constraining its amplitude to match a set of constraints corresponding to low‐resolution image measurements taken, and its spectrum to match the Fourier domain constraint.^8^, ^9^ Therefore, phase‐retrieval is crucial in Fourier ptychography as it is necessary to recover the phase information lost within the imaging process.

Early techniques that manipulated Fourier space used algorithms such as the Gerchberg–Saxton algorithm to conduct the phase‐retrieval process.^10^, ^11^ It effectively laid the groundwork for the possibilities of FPM but is computationally slow and often unsuitable for working with large image datasets.^3^, ^12^ In addition, the earlier algorithms used nonconvex techniques, rapidly increasing the computation rate with alternating dimensional phase‐retrieval methods.^13^, ^14^ However, these methods may not fully converge and add artefacts around the edges of the image (vignetting) or reduce the achievable resolution of the combined image due to increased noise.^3^, ^15^ Each algorithm used in FPM is unique for the area of research in focus. In modern FPM, algorithms can be generally classified into two categories: global and sequential gradient algorithms.^16^ Global gradient algorithms use the entire dataset of diffraction patterns to update the object function at each iteration. Whereas in the sequential gradient algorithm, the objective function is updated using diffraction patterns one by one. Several different phase‐retrieval algorithms are currently used in varying applications and data‐size requirements.

An additional benefit of using FPM is its unique ability to computationally correct aberrations of the objective lens of the microscope system. This is achieved by the ability of the method to constantly adapt to focusing errors and phase differences. Standard phase‐retrieval algorithms work by modelling the aberrations as a pupil function. For example, a second‐order pupil function can compensate for defocus aberrations.^17^, ^18^ The ability of ptychography to correct aberrations allows lower‐quality optics to be used, heavily reducing the setup cost and improving the ability of low‐cost microscopy tools to take high‐resolution images. There are also several practical benefits, such as a long working distance due to the low NA objective, speed, impact, and portability compared to other microscopy methods.^4^, ^19^

More advanced techniques use DL as it has succeeded in image reconstruction in FPM and other fields due to its ability to solve complex inverse problems. It has the potent to achieve results than current model‐based techniques.^20^ In addition, DL techniques have several practical benefits for image reconstruction problems due to lower computational costs and higher speed in comparison to iterative reconstruction algorithms. Techniques such as fluorescence, X‐ray, and ultraviolet (UV) microscopy have been adapted for use with FPM and use phase‐converging algorithms or machine learning to optimise the retrieved phase until convergence is achieved by minimising the loss function.^21^, ^22^, ^23^, ^24^ FPM is currently used in many digital pathology imaging modes, including multimodal, two‐ and three‐dimensional cell modelling, birefringent imaging, and camera scanning techniques.^3^, ^25^, ^26^ These techniques have increased in popularity due to improvements in the optical system, low‐artefact image quality and ease of use with current microscopes.^27^ The advancements in machine learning, specifically in iterative algorithms and DL convolutional neural networks (CNN), have also vastly improved Fourier ptychography medical imaging.^28^

This review introduces the fundamental concepts in Fourier optics, builds a fundamental understanding of Fourier ptychography (including phase‐retrieval algorithms used in FPM), and shows the rapid advancements in this approach since the advent of machine learning and deep learning algorithms. Advancements in algorithms have also been complemented by improvements to the experimental setup. This has led to the current digital pathology applications of FPM. In conclusion, we will discuss our perspective on the future of FPM, specifically within the field of digital pathology.

## 2 FOURIER OPTICS AND PTYCHOGRAPHY

FPM fundamentally relies on how an electromagnetic wave (EM) propagates through a medium. Fourier optics is used in various fields, from astrophysics to biological computational research. In this next section, wave propagation and resolution concepts (within the scope of FPM) will be introduced and developed into Fourier optics, focusing on FPM and its application in digital pathology.

### 2.1 The Abbe resolution limit

In any form of optical microscopy, the image is formed by the interference effect of plane waves propagating along different directions. Within the sample plane, the spatial frequency generates pairs of plane waves which propagate symmetrically to the optical axis. However, due to the diffraction of visible light wavefronts, there is a resolution limit as they pass through the aperture of the objective lens. As the wavelength decreases, the respective diffraction angle reaches a point where it exceeds a resolution limit allowed by the objective, called Abbe's diffraction limit.^29^ The formula for Abbe's diffraction limit is:

(1) $$\Delta x=\frac{\lambda }{2NA},$$

where, Δx, is the theoretical resolution of the system, λ, is the wavelength of light propagating through the system, and NA, is the numerical aperture of the objective given by:^30^

(2) NA=nsinα.

In the above equation, n is the refractive index, and α is half of the maximum angle of the cone of diffracted light captured by the objective lens. Assuming that the lens receives all of the possible light scattered by a sample of interest, it will be able to resolve distinct points of the same width as the optical wavelength in the system. However, the lens almost always accepts a smaller light cone, so its resolution is proportionally smaller than the theoretical resolution. To fix this, one could use an objective lens that agrees with a larger cone of light, that is, with a larger NA, but this typically results in a reduced field‐of‐view (FOV) and depth of field. Combined with computational algorithms, Fourier ptychography solves this problem through the use of synthetic apertures.^31^

### 2.2 Synthetic apertures and fundamental mathematical concepts

Synthetic apertures were first developed to bypass the resolution limit at radio wavelengths in telescopes. Astronomers used a relatively simple method of combining images from several telescopes and merging their phase and intensity information into a new, improved image.^32^ More recently, several more advanced physical and computational techniques have been used to overcome the physical aperture limit, building upon the early telescope method.

Synthetic apertures in microscopy have several benefits and allow higher‐resolution imaging despite the limitations of physical apertures in optics, due to the direct correlation between these and the amount of light collected for imaging. By optimising images from several angles and matching them in varying ways, the NA physical limit set by the objective lens is bypassed computationally, allowing enhanced sample resolution.^33^

FPM creates a synthetic aperture by combining physical principles in Fourier optics with computational imaging techniques. The standard method for FPM involves replacing the light source (theoretically regarded as a point source) used in standard microscopes with an LED array. The light from LEDs in the array illuminates the sample at specific angles, while the objective lens collects it after interaction. A digital camera then records low‐resolution images that are frequency‐matched using phase‐retrieval algorithms such as Gauss‐Newton, which are used to complete the full Fourier plane spectrum. The images are then Fourier‐transformed and put into an iterative phase‐retrieval algorithm. Spatial frequency data is then Fourier‐transformed back into the image format, which creates a large‐FOV, high‐resolve image. In the early stages of FPM, algorithms would estimate the aberrations within the optical setup and then use an iterative phase‐retrieval algorithm to reconstruct the high‐quality image; this groundwork is still being used in some cases today.

The original FPM algorithm used was the Gerchberg–Saxton approach. This iterative phase‐retrieval process aims to acquire the phase of a complex‐valued wavefront from two intensity measurements acquired in two different planes. A finite pupil size is used as a constraint in the Fourier domain, and the corresponding amplitude constraints (intensity measurements captured in the images) are applied in the image domain. This coincides with letting the phase evolve as each image is stepped through sequentially. The Gerchberg–Saxton method, a type of gradient descent, represents a natural way to solve phase‐retrieval problems by trying to minimise some cost function that describes the differences between actual and predicted measurements. The Gerchberg–Saxton algorithm is still used for rapid colour FPM for high‐throughput digital pathology.^34^, ^35^ Zhang et al. created an algorithm that expanded on the Gerchberg–Saxton principles and used the RGB wavelengths of LEDs to develop a fast diagnosis method using FPM. However, one critical hindrance which prevents the use of the original Gerchberg–Saxton is that its formulations are often nonconvex and lack global convergence guarantees.^36^ Therefore, when applied to more complex FPM techniques, the algorithm can break down, causing artefacts. The Gerchberg–Saxton algorithm is reliable for digital pathology where high throughput is critical. However, it has limits regarding images with a high resolution.

Recent advances in machine learning have allowed significant improvements and different techniques to be developed. Several published methods combine FPM with machine learning to optimise paths.^15^ There are two main types of machine learning optimisation: same‐order criteria, where the convergence of a path is ensured, and background criteria, which are used for local modifications of the FPM phase‐retrieval algorithm. The same‐order criteria effectively estimate the aberrations in the imaging system before the experiment and then correct them in the phase‐recovery algorithm. The background criteria aim to optimise the reconstruction algorithm directly to correct the imaging system aberrations without using an ansatz as part of the algorithm.^3^, ^15^, ^37^ Many phase‐retrieval algorithms for FPM in the field today use various methods, beginning with embedded pupil function recovery and building up to highly advanced algorithms. After the algorithm converges to a result, the phase‐matched frequencies are joined to reconstruct high‐resolution images. The theoretical modification of the standard Abbe's diffraction limit formula for FPM is given by:^3^, ^29^

(3) $$d_{x,y}=\frac{\lambda }{NA_{obj}+NA_{ill}},$$

where, $NA_{ill}$ is:

(4) $$NA_{ill}=n\frac{\sqrt{{\left(\frac{x_{m}-1}{2}\right)}^{2}z+{\left(\frac{y_{m}-1}{2}\right)}^{2}z}}{\sqrt{{\left(\frac{x_{m}-1}{2}\right)}^{2}z+{\left(\frac{y_{m}-1}{2}\right)}^{2}z}+h^{2}},$$

where $NA_{obj}$ is the numerical aperture of the objective lens, $NA_{ill}$ is the illumination aperture, $x_{m}$ and $y_{m}$ represent the number of LEDs in their respective dimensions, z is the distance from the LED to the sample and h is the distance between the LED used and the optical axis. The main goal of allowing high‐resolution imaging is to find an optimal point spread function for the object, which models the intensity distribution imaged by the camera under different angle illuminations. The iterative algorithms are therefore used to estimate the point spread function, introduce constraints, update the object function spectrum, and repeat this process until it converges at a minimum distribution for the spatial frequency. Hessian matrices are critical when fully understanding the phase retrieval process; they are used to search for minimisation functions that will improve the convergence speed of the algorithm. The Hessian matrix is a square matrix of second‐order partial derivatives of a scalar‐valued function. Hessian matrices are often used in machine learning and algorithms to optimise a mathematical function of interest by describing its local curvature. Suppose we have a function (f) of n variables; the Hessian of f is given by the matrix;

(5) $$H_{f}=\left[\begin{array}{cccc} \frac{\delta^{2}f}{\delta x_{1}^{2}} & \frac{\delta^{2}f}{\delta x_{1}\delta x_{2}} & \cdots & \frac{\delta^{2}f}{\delta x_{1}\delta x_{n}}\\ \frac{\delta^{2}f}{\delta x_{2}\delta x_{1}} & \frac{\delta^{2}f}{\delta x_{2}^{2}} & \cdots & \frac{\delta^{2}f}{\delta x_{2}\delta x_{n}}\\ \vdots & \vdots & \ddots & \vdots \\ \frac{\delta^{2}f}{\delta x_{n}\delta x_{1}} & \frac{\delta^{2}f}{\delta x_{n}\delta x_{2}} & \cdots & \frac{\delta^{2}f}{\delta x_{n}^{2}} \end{array}\right].$$

The diagonal elements within the matrix represent the second‐order partial derivatives concerning each variable $x_{n}$. These elements show how the function's curvature varies with the function's rotation of each variable. When using an iterative optimisation algorithm, Hessian matrices are used to determine the gradients of the intensity‐based cost functions, which aim to minimise the difference between the estimated and measured intensities, increasing the convergence rate.^38^

The Wirtinger gradient descent is critical for optimising functions with complex variables and is, therefore, especially useful in Fourier ptychography and is used in many areas of FPM. It is a modification of the traditional Newton gradient descent aimed at handling functions with both real and complex numbers. The Fourier transform of an image involves both real and complex components. The goal of phase‐retrieval algorithms is to optimise these functions to allow them to solve inverse problems accurately and efficiently. The Wirtinger gradient descent uses Wirtinger derivatives to compute gradients in the complex domain:

(6) $$\frac{\delta f\left(z\right)}{\delta z^{*}},\frac{\delta f\left(z\right)}{\delta z},$$

where, $z^{*}=x-iy$ is the complex conjugate of *z*, $\delta f\left(z\right)/\delta z^{*}$ is the derivative of the function $f\left(z\right)$ with respect to the complex conjugate of *z*, and $\delta f\left(z\right)/\delta z$ is the derivative of $f\left(z\right)$. The derivatives allow mathematical analysis of the function's real and imaginary parts in a computationally efficient way. The gradient of the objective function is computed for the complex variables, which are then updated to the direction of the negative gradient to minimise the function.^39^ In FPM, the aim is to synthetically create a high‐resolution image by solving an inverse problem. Inverse problems involve estimating the complex wavefront of the object from a series of low‐resolution measurements, which give intensity information. However, the phase information is still required for reconstruction. Wirtinger gradient descent is used to iteratively update the estimate of the ansatz complex wavefront based on the difference between the observed and estimated intensity patterns in the Fourier domain.^40^

A critical step in the advancement of FPM was the embedded pupil function recovery (EPRY) algorithm. The algorithm allowed recovery of the pupil function and the Fourier spectrum simultaneously, eliminating the previous requirement of knowing the aberration in the imaging system.^41^ This meant that FPM no longer required the time‐consuming acquisition of pupil characterisation data, drastically improving the speed of FPM. The method begins with an ansatz of the pupil function and the sample spectrum, which is set as a circular‐shaped low‐pass filter with uniform zero phase (only ones inside the pass band and zeros outside the passband). The passband radius is 2πNA/λ (simulating the captured light from the aperture). The initial low‐resolution image is Fourier transformed and used as an initial sample spectrum guess. The images are then sequentially captured and convoluted with the pupil function and sample spectrum. This process is iteratively repeated, updating each loop. The EPRY‐FPM algorithm measures the real pupil function phases by accurately separating the pupil function from the sample spectrum. To highlight the success of this algorithm, images of a USAF target at varying FOVs were compared between uncorrected, standard correction and the EPRY‐FPM. In the pictures, a clear distinction in resolution quality was highlighted.^41^ However, the algorithm is sensitive to noise within the system, and the acquisition time is longer than other algorithms.^16^ Therefore, when imaging in noisy environments, the EPRY method on its own has downsides, and other techniques are recommended in combination with EPRY.

## 3 CURRENT DIGITAL PATHOLOGY APPLICATIONS OF FPM

**FIGURE 2:**
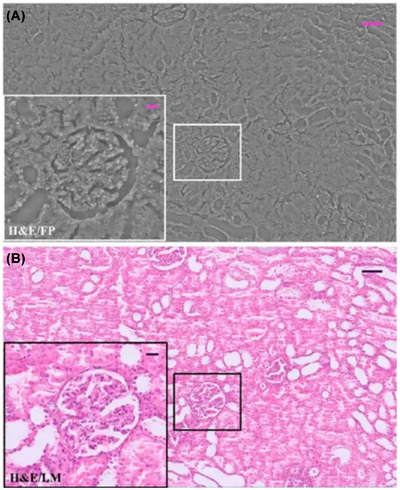
Haematoxylin and eosin stained kidney tissue. (A) A whole renal tissue image taken using FPM. (B) An image taken using standard light microscopy as a control comparison. The scale bar in the larger images is 125 μm and in the ROIs it is 25 μm (figure used with credit to Valentino et al.^42^ DOI: 10.3389/fphys.2023.1120099).

Since its development in 2013, Fourier ptychography techniques have evolved to allow faster imaging acquisition, full‐colour imaging, polarisation and fluorescence imaging. Digital pathology has rapidly expanded with the advancement of technology in the 21st century. FPM allows fast (ms per image), full‐colour, high‐resolution, extensive FOV imaging (${\mathrm{mm}}^{2}$ scales), and, therefore, has been used in whole slide scanners to uncover vast amounts of biological information.^24^, ^44^, ^45^ As mentioned, Fourier ptychography captures the intensity and phase of the image. FPM has been successfully compared with light sheet microscopy as a baseline in digital pathology. One study shows that it can be used in conjunction with digital interference contrast microscopy to allow physicians to diagnose kidney tissues successfully (as shown in Figure 2).^42^

**FIGURE 3:**
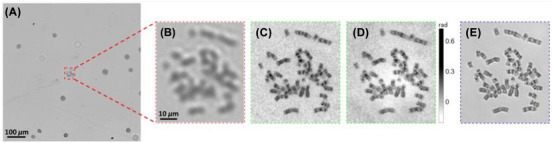
Single chromosome sample images from the blood sample of leukaemia patients. (A, B) were imaged using a 4×/0.1NA objective lens. (C, D) however were imaged using a 4×/0.1NA objective lens and reconstructed to get the amplitude and phase, respectively. (E) was imaged using a 20×/0.4NA objective lens, allowing comparison of the reconstructed images (figure from Zhang et al.).^43^

Other studies further highlight how FPM has allowed new insights in digital pathology: the study of circulating tumour cells (CTCs) and chromosomes (see Figure 3 for results in the chromosome analysis).^43^, ^46^ CTCs are a valuable biomarker with strong potential in improving prognosis and diagnosis of recurrence. They are identified and isolated using microfilters fabricated with a defined pore size, as CTCs are larger than red blood cells (RBCs).^47^ One severe limitation in this process for analysis is that after sample processing, the surface of the microfilters becomes uneven, causing captured CTCs to be in varying focal planes.^48^ This makes it difficult for standard microscopy techniques. Williams et al. have used FPM to propagate the field to different focal depths in the ptychography process, allowing different sample regions to be brought into focus, significantly reducing the acquisition and analysis time.^46^ In the study of chromosomes, Zhang et al. found that FPM methods could resolve the chromosome samples better than a light microscope (detailed in Figure 3).^43^ In addition, their work led to a new strategy for efficient slide scanning for chromosome karyotyping, acquiring the raw images using a 10× objective lens and then reconstructing the high‐resolution, analyzable metaphase chromosomes for digital pathology.

This section details modern FPM and describes the specific algorithm required for each technique and/or wavelength. Finally, we highlight how they can be used for digital pathology.

### 3.1 Multiplexed illumination

Multiplexed coded illumination is used to optimise the sequential scanning process of the LED array in FPM. There are two main principles: taking the same number of images while reducing the exposure time for each, or reducing the total number of images taken during the process. The first method involves using the same sequential process of taking images but turning on more LEDs, which will increase the light throughput.^49^ A strong irradiance from the object is created by illuminating the object from many light sources, which leads to clear and bright images. Assuming the random patterns are linearly independent, the taken images can be interpreted as a linear combination of images from each LED. Therefore, the data should contain the same information as in a standard sequential scan.^50^ The acquired images are then decoded and analysed on the computer. This method has several advantages in that the acquisition time is significantly less, and the signal‐to‐noise ratio (SNR) remains high due to multiple sources for the image. However, the SNR is assumed to be better when compared to single‐source illumination, considering that the images acquired are not overly saturated. At the saturation limit, multiplexing should be avoided, as when the number of sources of light increases, the SNR will decrease. In addition, the number of sources can only increase at the cost of single‐frame exposure time. The second method of multiplexing significantly reduces the data requirements by using a random coding strategy to take fewer images. Using a fixed number of LEDs, Tian et al. opted to randomly vary the location of the LEDs while ensuring each LED is used at least once in each dataset.^2^ The first image in each dataset effectively chooses which LEDs to turn on randomly and then follows a few simple rules aimed at excluding LEDs which have already been used. By doing this, each image will contain information from multiple areas of the sample's Fourier space, spanning the entire area of LEDs. The method allows good information mixing while maintaining a balanced, nonoverlapping coverage of Fourier space for each image. However, the quality of the reconstructed high‐resolution images degrades linearly with the amount of multiplexed LEDS.^51^ This technique inspired the development of colour multiplexed coded illumination. The colour FPM method uses three‐channel images corresponding to the RGB LEDs, separated by an image colour sensor, which acts as a phase input for recovery in the algorithm. The plane wave is emitted, and assuming every single colour is spatially coherent after passing through the optical system, the plane wave is a combination of several monochrome exit waves.^52^, ^53^ Sun et al. have used multiplexed colour illumination to obtain in vitro images of HeLa cell mitosis.^50^ Their work theorised that in the future, their method could be used for many applications, including digital pathology.

Multiplexed coded illumination is a key method in advancing FPM. It significantly reduces the acquisition time while maintaining a good resolution and large FOV. Combining this with other FPM imaging setups is critical for FPM's use in digital pathology.

### 3.2 Colour FPM

Acquiring accurate colour images of pathology slides rapidly is critical to the advancement of medicine. Colour FPM has been proposed as a solution for high‐throughput digital pathology. In the earlier developments of colour FPM, the process achieved high‐resolution, large FOV images by separately reconstructing high‐resolution images from the individual red, green and blue (RGB) channels and then combining the images. However, this process suffered from a lengthy acquisition time, and the individual errors in each of the three channels would combine, causing poor reconstruction.^34^ More methods use multiplexing and colour transfer techniques but suffer from long acquisition time and difficulty matching the reconstructed and original image colours, respectively.^15^, ^35^, ^54^

Several methods have emerged which vastly improve the acquisition speed.^52^ Zhang et al. have proposed a fast‐colour FPM imaging technology with fusion colour correction (CCFPM).^34^ The process works by acquiring the greyscale image of the green channel and iteratively reconstructing it using the Gerchberg–Saxton algorithm. They demonstrated that the adapted Gerchberg–Saxton algorithm has a computation time of less than 1 s, reduces the chromatic aberrations, and consistently returns high‐quality reconstructed images.^34^ Chen et al. developed a novel colour FPM method, which is a further improvement in speed in comparison to the standard colour FPM with a marginal increase in the root‐mean‐square error and a reduction in the number of artefacts caused by dust, etc. within the system.^15^ Colour FPM is a promising method for advancing digital pathology; however, problems with colour leaking and/or data acquisition time need further improvement for clinical use. In addition, techniques using algorithms and neural networks tend to be computationally intense, reducing their ability for clinical use. Therefore, methods such as CFPM have not fully utilised the potential of colour transfer and need improvement in quality and computational efficiency.

**FIGURE 4:**
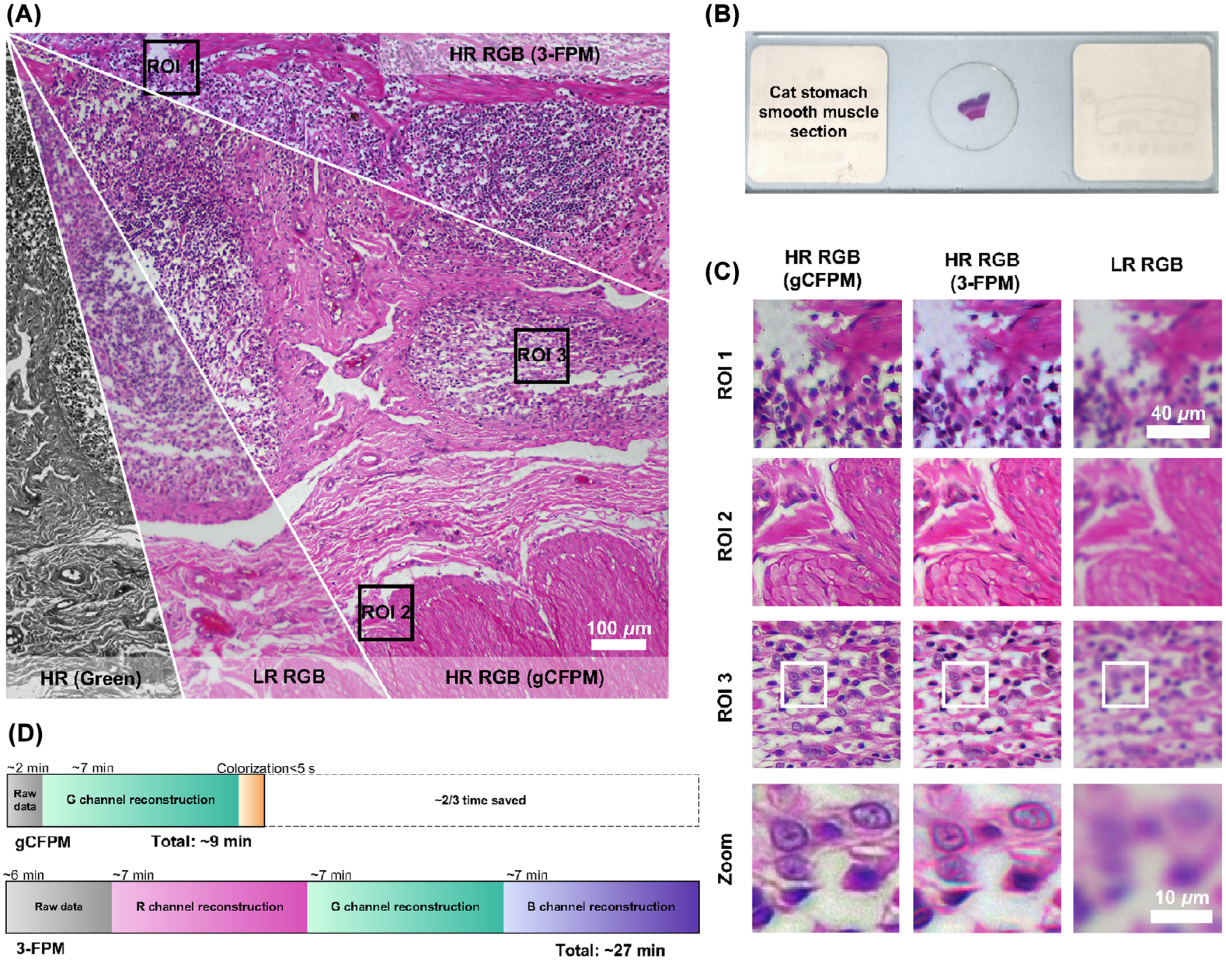
Full‐colour imaging of a cat's stomach muscle sample via gCFPM. (A) The overall imaging result of the sample. (B) The smooth muscle section. (C) An enlarged view of each ROI. (D) A full process time comparison of three‐channel FPM and gCFPM. As illustrated in Chen et al.^55^

More recently, Chen et al. have proposed a new method allowing fast full‐colour pathological imaging using FPM called gCFPM.^55^ Their work is based on a generalised dual‐colour‐space‐constrained model, where the colour transfer problem mentioned in other methods is addressed from an optimisation standpoint. General CFPM typically transfers colour in RGB (a single colour space), which leads to artefacts created due to poor spectral matching. In gCFPM, two complementary colour spaces are used while being matched simultaneously, constraining the solution to avoid artefacts. This enables non‐iterative and precise colourisation using only a low‐resolution colour image and a single‐channel FPM reconstruction. For megapixel images general CFPM will take approximately minutes to hours depending on the size and amount of images, whereas gCFPM is recorded to be up to 3× faster (approximately 3 s) and has the added benefit of removing artefacts, while achieving a resolution of 5000 × 5000 pixels (see Figure 4 for details on processing time). Their work has clear potential in digital pathology, and they show this through imaging of cat stomach muscle and then comparing the image quality with previous methods. Therefore, gCFPM addresses several of the problems, such as colour quality and computational time and will likely be an invaluable and leading technique for the future of colour FPM for digital pathology.

### 3.3 Polarisation FPM

Polarisation microscopy is an established technique commonly used to observe birefringent effects in samples. Polarisation Fourier ptychography microscopy (pFPM) expands upon the principles used in polarisation microscopy and Fourier optics to allow large‐area, large FOV, high‐resolution, birefringence imaging. The key difference between pFPM and standard polarisation microscopy is that a circular polariser is used for light reaching the sample instead of a linear polariser. The reason for this is that at high illumination angles, the electric field of a plane wave begins to diffract and is no longer exactly transverse to the optical axis. The linear polarisation vector effectively splits into a p‐component (in the plane of incidence) and an s‐component (perpendicular to the plane of incidence), where their relative intensities change with tilt angle, that is, different LEDs affect the polarisation intensity of the sample. However, when using a circular polariser, the electric field has equal p and s amplitudes. Therefore, the total intensity travelling through the objective remains constant when illuminating at high angles, and it is easier for standard algorithms to reconstruct the images.^56^ However, it is worth noting that although more difficult, Dai et al. have successfully adapted an algorithm to allow a linear polarisation setup (more detail in Section 3.3.1).^57^

The image recombination process in pFPM builds upon fundamental principles of polarised light described by the Jones matrix (first proposed in 1944).^58^ The Jones matrix formulation of polarised light comprises 2 × 1 Jones vectors to define the field components and 2 × 2 Jones matrices to describe polarising components. The Jones matrix assumes that the propagating light within the system is fully polarised and cannot fully describe unpolarised or semi‐polarised light. The Jones vector contains different phase factors for each vector component, providing a compact description of a homogeneous monochromatic harmonic wave. The 2 × 1 Jones column matrix is:

(7) $$E\left(0\right)=\left[\begin{array}{c} E_{x}\\ E_{y} \end{array}\right]=\left[\begin{array}{c} E_{x}exp\left(j\delta_{x}\right)\\ E_{y}exp\left(j\delta_{y}\right) \end{array}\right].$$

The Jones matrix for a nondepolarising thin specimen is:

(8) $$J_{s}=RDPR^{-1},$$

where R=[cosθ−sinθ;sinθcosθ] is the rotation matrix defining the optic‐axis orientation. D is the diattenuation matrix and $P=\left[e^{i\delta /2}\;0;\;0\;e^{-i\delta /2}\right]$, wherein δ is the phase retardation.^59^

**FIGURE 5:**
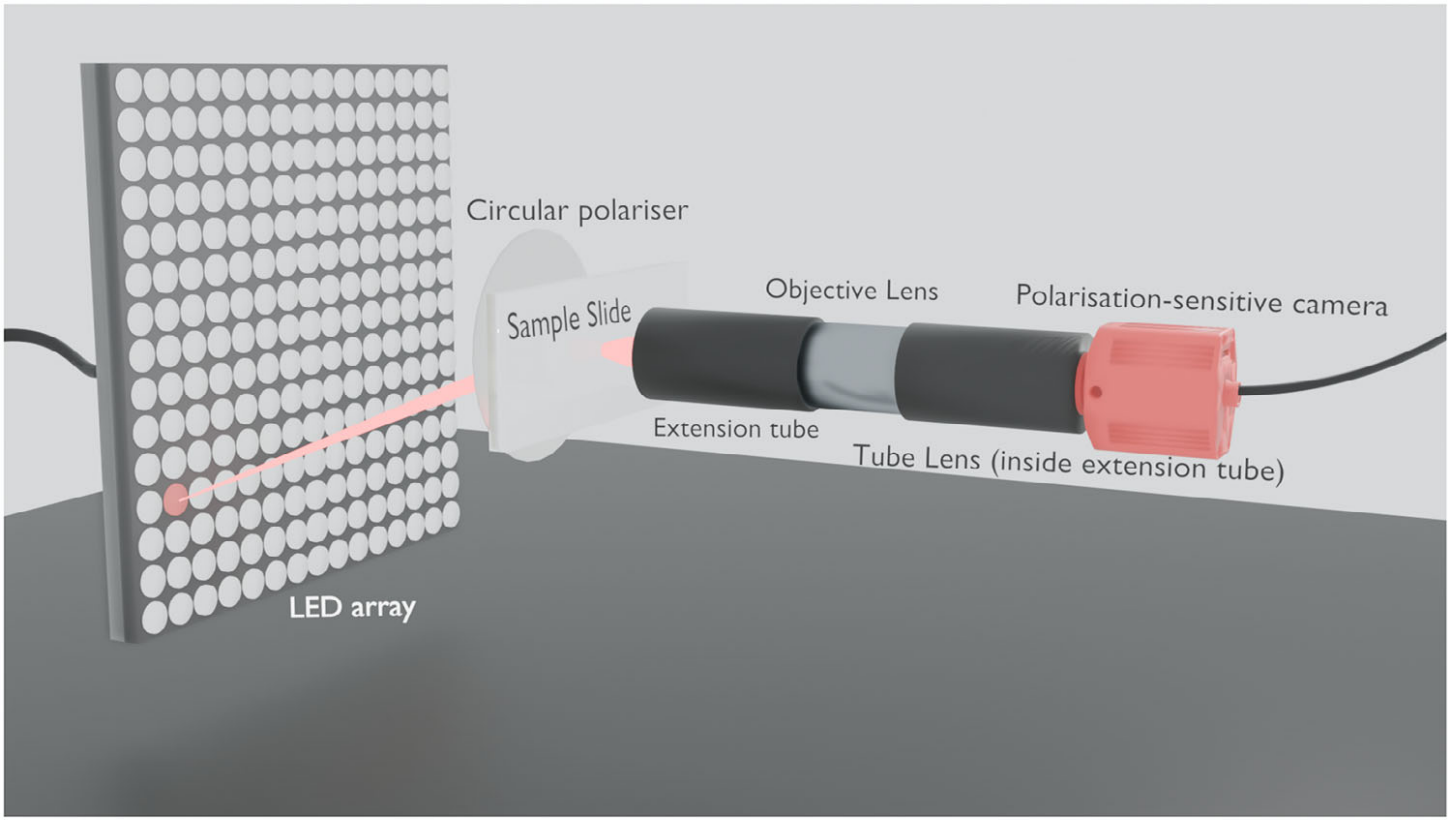
A modified version of the FPM setup is adapted by adding a circular polariser between the LED array and the sample and a polarisation‐sensitive camera. As illustrated, this modification will allow polarisation‐resolved images of anisotropic biological cells.

The 2 × 2 formulation is relatively straightforward compared to the Mueller matrix formulation and describes pFPM more accurately as it assumes the sample is illuminated by completely polarised light. In pFPM, an LED array still provides angularly varying light. However, there is a crucial difference for pFPM compared with the two imaging states (brightfield and darkfield) in standard FPM; the four polarisation states ($0^{\circ },45^{\circ },90^{\circ },135^{\circ }$) must be accounted for.^56^, ^60^ Several polarisation angles/states are necessary for exhibiting the varying effects in analysing anisotropic media. Standard pFPM allows this by adding a circular polariser below the sample slide and a polarisation array to the camera to enable polarisation sensitivity from the four angles (as shown in Figure 5). The critical component in the image capture process will be the intensity measurements within each image taken, which is key for highlighting the anisotropic specimens within the sample.

To allow pFPM, algorithms such as the Gerchberg–Saxton and EPRY have been adapted and improved for imaging varying anisotropic samples.

The pFPM process requires four different datasets/imaging stages corresponding to the four polarisation states in pFPM. Therefore, it is necessary to reduce the setup capture time. Several researchers have developed custom hemispherical LED arrays to overcome problems with LED spacing and depth of field imaging and create benefits such as reducing the required LEDs.^26^ By using a planar LED board, the NA is effectively limited, and by increasing the incident angle, the signal‐to‐noise ratio (SNR) decreases due to the dark field containing more noise.

**FIGURE 6:**
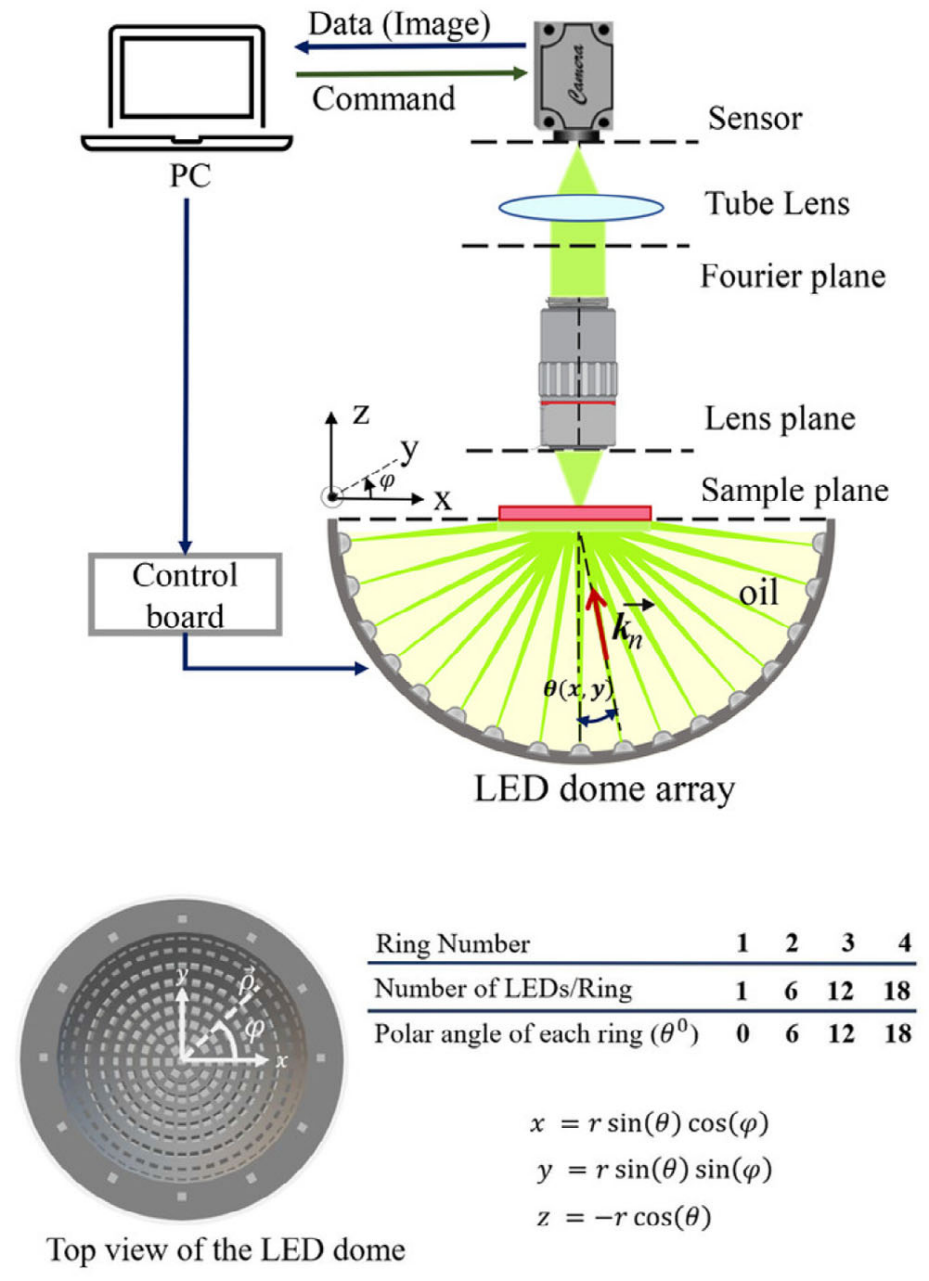
Diagram detailing the dome‐shaped LED array, which is used to (1) reduce the required number of LEDs; (2) improve LED spacing; and (3) address common depth of field imaging challenges in FPM (figure sourced from Mayani et al.^26^).

A dome LED array has been suggested to improve these shortcomings. An essential requirement for successful imaging is that Abbe's diffraction limit must be satisfied for the dome array, that is, the object spectrum from the adjacent rings must overlap by more than 50%. This will reduce many problems, such as aberrations and acquisition time and help reduce the postprocessing time. A dome array has three benefits: the number of LEDs used is significantly less, a higher NA is achieved and better illumination when at high angles (dark field imaging), achieving an improved SNR compared to a standard planar LED array (see Figure 6 for an example of a dome‐shaped array).^26^, ^56^ An example of the dome‐shaped being used for digital pathology is Zhou et al., who have used a quasi‐dome design to obtain high‐resolution, large FOV images of a mouse heart slice.^61^ Mayani et al. have used a dome‐shaped area in combination with the EPRY algorithm to iteratively retrieve the phase and improve the convergence while maintaining the aberration correction.^56^ In addition, their work was shown to measure specific anisotropic properties of transparent samples such as liver tissue for diagnosis of liver fibrosis stages, achieving a resolution of 244 nm and a synthesised NA of 1.1 compared to the objective NA of 0.28.

#### 3.3.1 Vectorial pFPM

**FIGURE 7:**
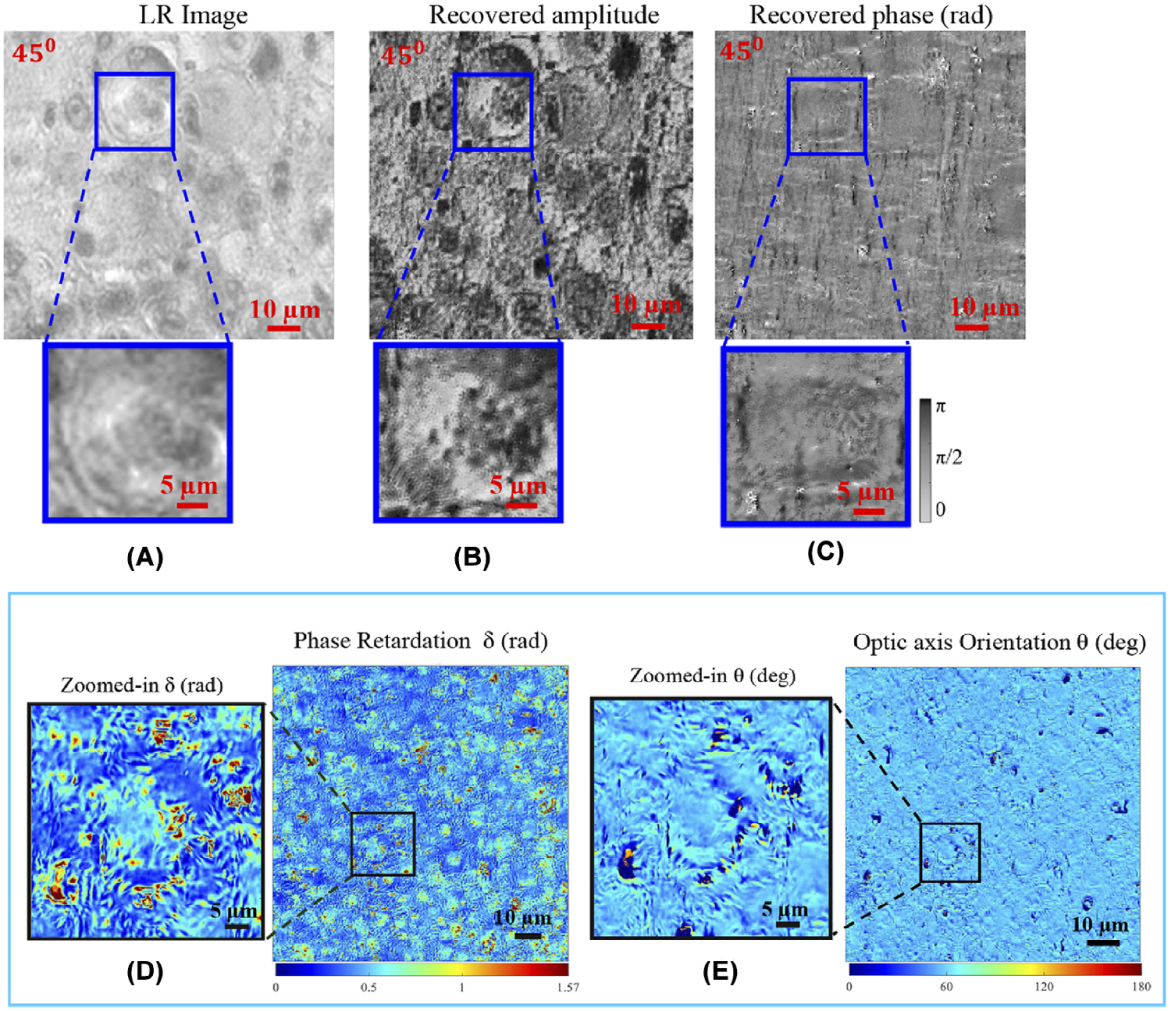
Images of liver fibrosis used for determination of liver fibrosis stage, testing pFPM's potential in digital pathology: (A) The captured low‐resolution interpolated image. (B) The recovered amplitude. (C) The recovered phase. (D) The measured phase retardation (δ). (E) The optical axis rotation (θ). Using these images, a pathologist successfully diagnosed the stage of liver fibrosis in the patient, showcasing the potential for pFPM in digital pathology (figure originally published in Mayani et al.^56^).

Another example of pFPM being used for digital pathology is vectorial Fourier ptychography, an advanced polarisation FPM technique that expands upon the Gauss‐Newton algorithm (first proposed to improve FPM by Yeh et al. in 2015).^38^, ^62^ Their work expanded upon the Newton algorithm by incorporating adaptive machine learning to narrow the convergence further. Through approximation and unrolling, the Gauss–Newton method reduces the computational intensity and is even argued to be more accurate than the traditional Newton and Gerchberg–Saxton methods.^38^ Neural networks have since been combined with vectorial FPM to recover complex object information via a network training process.^63^ The method begins by modelling the input of the CNN as a coherent transfer function in the Fourier domain or a point spread function in the spatial domain.^64^ It then iterates to measure the difference between the prediction and the actual measurement (using similar principles to Wirtinger derivatives).^39^ The network output is then modelled as a loss/cost function, minimised until the results converge. This advancement is critical and has been focused on applications such as cell imaging and to allow diagnosis of liver fibrosis, highlighting its ability to be used in digital pathology (shown in Figure 7).^42^, ^56^ Dai et al. proposed an advanced pFPM method using vFPM to improve the iterative reconstruction algorithm to incorporate fewer approximations to the Jones' matrix.^57^ The vFPM algorithm also accounts for polarisation‐dependent imaging systems, advancing a relatively novel field in microscopy. To demonstrate the performance of vFPM, which is capable of large‐area (29 ${\mathrm{mm}}^{2}$), high‐resolution (1240 nm full‐pitch) reconstructions. Cardiac tissue was used for plaque detection, highlighting its potential for digital pathology.^65^, ^66^, ^67^ This technique also has an additional benefit in correcting birefringent systematic errors caused within plastic lenses.^57^ Therefore, it can be used to improve low‐cost setups used to analyse anisotropic specimens. With improvements in the vFP algorithm and incorporating methods such as multiplex‐coded illumination, vFP has clear potential for use in digital pathology in non‐noisy environments.^68^

The algorithm was also combined with the LED dome area for pFPM to analyse liver tissue. Another critical point the research highlighted was the benefits of this algorithm, which reached beyond FPM and had potential use in general optical system aberration correction. Their work demonstrated that the stage of liver fibrosis can be determined using pFPM. Liver fibrosis is difficult to detect, yet early diagnosis is essential to provide the patient with specific therapy to prevent progression to liver cirrhosis.^69^ Polarisation FPM has also been used to image several other biological specimens such as dog oesophagus slices (using multiplexing pFPM), dog tongue slices and mouse eyes.^70^, ^71^, ^72^ The main drawback of the dome‐shaped LED array is that calibration is significantly more difficult, which hinders its ability to be used in clinical digital pathology. Unfortunately, this is a common problem across all unique LED arrays, as it is often necessary to calibrate each LED illumination individually.

#### 3.3.2 Optical and intensity diffraction tomography

Originally, Fourier ptychography was only applicable to thin samples due to the wave properties emitted by the LED array when capturing properties of the sample's shifted optical spectrum. Optical diffraction tomography (ODT) is a promising method of achieving noninvasive quantitative high‐resolution imaging, first proposed by Wolf in 1969.^73^ ODT has been used in several research areas to allow 3D structure imaging.^74^, ^75^, ^76^ The similarities between ODT and FPM were shown in 2014 when Dong et al. first demonstrated a diffraction technique using a single plane wave to illuminate the sample, which allowed 3D refocusing while maintaining the benefits of FPM.^77^ Their method illuminated the sample using a single‐plane wave and linearly scanning the aperture at the Fourier plane of the optical system, acquiring the corresponding intensity images of the object. This setup bypassed the need for a thin sample in the early advancements of FPM. Like standard FPM, the acquired intensity images are then Fourier transformed and synthesised in the frequency domain acquired by the optical wavefront. The critical difference in this method is that the optical wavefront recovered (used to achieve high resolution) depends on how the wavefront exits the sample rather than how it enters it. By doing this, how the wave propagates through a thin versus a thick sample is irrelevant, as the recovered phase is after exiting the medium. Once recovered, the complex wavefront is back‐propagated to a plane along the optical axis for 3D holographic refocusing.^78^ However, the main limitation of this method is the requirement for mechanical scanning, limiting its use for high‐throughput applications. Another slight limitation is that the requirement for coherent illumination limits the approach from LED arrays and environments with ambient light.

Recently, Song et al. have developed polarisation‐sensitive intensity diffraction tomography (PS‐IDT).^79^ This novel form of 3D polarisation microscopy can reconstruct the Jones matrix of an anisotropic object from both weak and multiple scattering specimens from multiple intensity‐only measurements. Their method illuminates the object using circularly polarised plane waves at various illumination angles to encode the isotropic and anisotropic structural information into 2D intensity information. Their process was used to measure multiple specimens successfully and had a lateral resolution of 0.54 μm across a FOV of 80 μm × 80 μm. However, the setup has a relatively long acquisition time, preventing it from doing live‐cell imaging. They mention utilising the previously mentioned dome‐shaped array, which would improve the imaging speed, and by incorporating DL methods, the acquisition process could be improved in future work.

**FIGURE 8:**
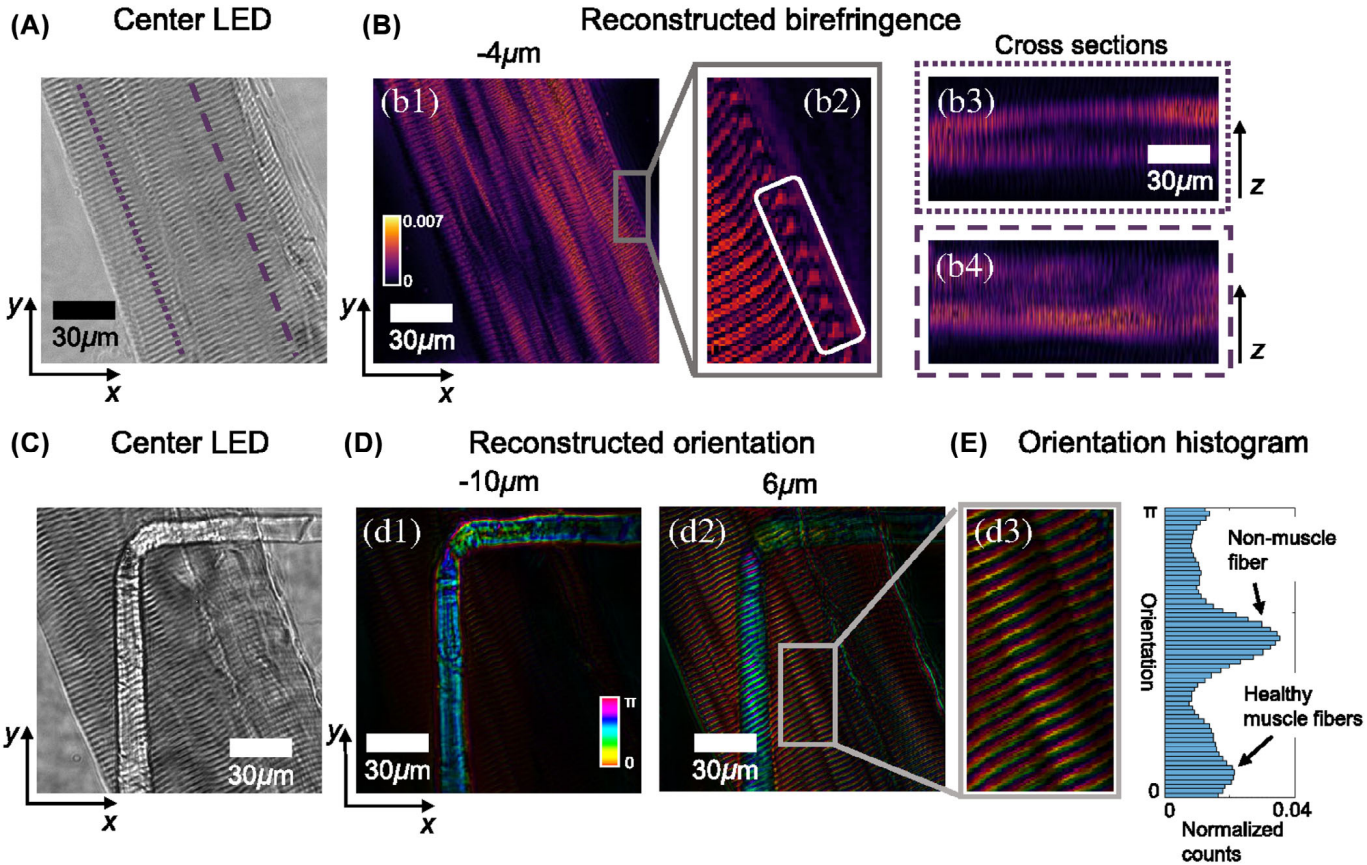
Pictures exhibiting the successful reconstruction of muscle fibre using $T^{2}oFu$. (A) The image taken using illumination from the centre LED focused on the muscle fibre. (B) The reconstructed birefringence, with the ROI showing the structures of healthy muscle fibres. (C) The image was taken after focusing the imaging system between a non‐muscle fibre with a 90‐degree bend and the same muscle fibre at a different region. (D) The reconstructed orientation at different depths, with a zoomed‐in view showing the fine sarcomere structure of muscle tissue. (E) Histogram of reconstructed orientation comparing healthy and non‐muscle fibres shown in D (figure from Xu et al.^80^).

Another example of diffraction tomography being used in 3D imaging is the work presented by Shiqi Xu et al. using tensorial tomographic Fourier ptychography ($T^{2}oFu$) for muscle tissue imaging (as shown in Figure 8).^80^ $T^{2}oFu$ is a nonscanning label‐free tomographic 3D microscopy method used to examine quantitative phase and anisotropic specimen information simultaneously. Similar to other tomography techniques, it expands upon the principles of FPM by incorporating the vectorial nature of light while using the quantitative phase information achieved via FPM. Their method had sufficient resolution to resolve cardiac amyloidosis (a lethal disease estimated to affect over 74,000 people worldwide).^81^ In addition, their method successfully identified changes in myofibrillar organisation, which can lead to skeletal myopathies, and therefore, rapid detection through high‐contrast and high‐resolution structural imaging is invaluable.^82^

Recovering the quantitative phase information is critical for obtaining accurate image reconstructions in FPM. Spatially coded Fourier ptychography has been used for true quantitative phase imaging, where a flexible and detachable coded thin film is attached across the image sensor in a standard FPM setup.^83^ The coded thin film contains microparticles, which spatially encode the complex wavefronts of the object, converting the phase information into detectable intensity variations. Through analysis of the spatial‐frequency contents of the input ground‐truth phase and recovered phase images, a uniform phase transfer function is obtained. In standard FPM, a nonuniform phase is normally recovered, leading to a constrained accuracy on the phase recovery. This technique demonstrates (through imaging of biological samples) the potential of spatially coded Fourier ptychography to bypass this historical constraint and aims to expand the application to Fourier ptychographic diffraction tomography.

Zhou et al. have developed a novel method combining transport‐of‐intensity with Fourier ptychographic diffraction tomography for 3D microscopy.^84^ The transport of intensity equation is one of the most famous approaches for phase retrieval and quantitative phase imaging. It is used to recover the quantitative phase distribution of an optical field by through‐focus intensity measurements.^85^ Using this technique, they managed to resolve line profiles across the boundary of the cytomembrane in C2C12 cells and other subcellular organelle structures in HeLa cells, verifying a resolution of 254 nm (using 754 images captured within 2.52 min/6.29 min with dry/oil‐immersion objectives). Their work exhibits another method for achieving noninvasive 3D biological specimen imaging.

ODT and intensity diffraction tomography can be applied to FPM for several biomedical and digital pathological applications. It is still relatively novel and requires further development in the algorithms used (reducing the image acquisition time) for it to be used in clinical digital pathology.

### 3.4 Deep learning

In recent years, DL has been used for a wide range of image reconstruction methods, ranging from medical applications in computerised tomography (CT) and magnetic resonance imaging (MRI) scans to research applications in electron tomography and FPM.^86^, ^87^, ^88^ It has been used in several fields of medical research, such as DL microscopy, classifications of diseases, MRIs, fluorescence microscopy and prediction of high‐risk and low‐risk patterns of developing cancer in the future.^22^, ^88^, ^89^, ^90^ DL methods typically require significant datasets (approximately 5000–20,000 synthetic images, for example, simulated phase/intensity patterns and 30–1000 experimental images from FPM); therefore, reducing the reconstruction quality and processing time is essential.^37^, ^45^, ^91^ This subsection details the two main uses of DL in FPM: improving image reconstruction and image analysis for pathology.

#### 3.4.1 DL to improve image reconstruction in FPM

DL has been used to improve image reconstruction in FPM. By training DL neural networks to eliminate defocusing and reinforce contrast within the images, a virtual *z*‐stack can be reused in further deep neural network (DNN) training.^45^ Enabling a further improvements in DL‐FPM by reducing the required dataset and therefore reducing the image acquisition time, while continuing to improve the reconstruction quality.

Another example of DL being used to improve FPM is in pFPM, where it has been used to improve convergence within the algorithm and continuously modify the phase within the algorithm, increasing the reconstruction accuracy.^42^, ^64^

Recently, DL is being combined with FPM for live‐cell imaging, through better analysis of the spatial and temporal information. Nguyen et al. have shown it was possible to reconstruct high‐SBP videos using a CNN trained solely on the first FPM dataset captured at the start of a time‐series experiment.^37^ Their method successfully reconstructed a high‐resolution (12,800 × 10,800 pixel) phase image in approximately 25 s (after training the network, which was approximately 16 h), while reducing the required number of images (173 low‐resolution intensity images) in each time window by 6× compared to standard FPM. Their method focused on solving the two main problems to solve when imaging live cells: independent datasets from static objects and sequential datasets from dynamic objects.^37^ To solve the independent problem, unique input‐output pairs are obtained by repeating the same image process and are then presented to the CNN to optimise the network's parameters. In sequential problems, the temporal correlation of a dynamic process requires additional information to be recorded using video datasets.^28^ Their method addresses both issues and simplifies them by assuming that in any live cell experiment without precise cell synchronisation, at any given time, there are samples covering all cell states. They then train the CNN using a single frame from the FPM, before showing that it can successfully reconstruct large‐SBP phase videos of time‐series live cell experiments.

Improving phase analysis and therefore, image reconstruction is essential for FPMs use in digital pathology. Methods improving the image acquisition process quality, while reducing the dataset is a fundamental improvement for clinical digital pathology. The ability for DL to allow live‐cell imaging FPM will have profound implications for FPM in digital pathology.

#### 3.4.2 DL for image analysis in FPM

Typically, an individual layer of a CNN is composed of a convolutional layer and a nonlinear operator. The filters (kernels) in these convolutional layers are randomly initialised and can then be trained to perform specific tasks through monitored or untutored machine‐learning techniques.^92^ In most imaging‐related problems, the neural network is fed several images, and the CNN is trained to identify unique characteristics, which are then assigned a numerical value. The extracted features from the image are then used as input predictor variables to a classifier, and a predictive model is formed.^93^, ^94^ The predictive model is then adapted using statistical weights of the unique characteristics. These are based on properties within the training images that determine if the taken image belongs to one of the training sets. However, the effectiveness of the feature descriptors used in CNNs strongly depends on the domain expertise of the developer.^89^ The capability to assign image characteristics to numerical values allows mathematical formulations and empirical image analysis. This can lead to problems such as false positives or negatives if the model is not trained correctly.^95^ Another problem is the reliance on large datasets to train the CNN correctly.^37^ Therefore, new imaging techniques need a longer proving time to develop an extensive database for training CNNs.^96^

DL has been used to develop and enhance the FPM process further, improving image quality and potentially leading to faster recognition of diseases in images by AI. By continuing to train and adapt DNNs, digital pathology will advance rapidly, leading to faster diagnosis and better research insight.

### 3.5 Fluorescence and UV Fourier ptychography

**FIGURE 9:**
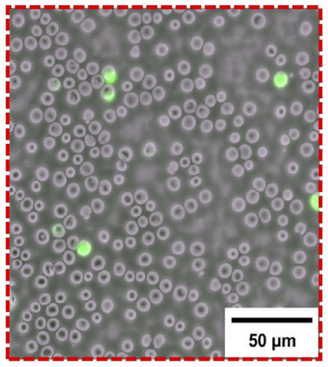
Overlayed quantitative phase and fluorescence images taken using FPM. Red blood cells in the phase image, which overlapped with the measured fluorescence (shown in green), were labelled infected, whereas the remaining red blood cells were labelled as uninfected. Once labelled, the images of cells are then used for training machine learning models to generate predictions based solely on the quantitative phase data (figure used with credit to Akcakir et al.^22^).

Another example of FPM being used in digital pathology is fluorescence FPM, which can be used to automate the detection of malaria parasite infection on stain‐free blood smears (shown in Figures 8 and 9).^21^, ^22^ Akcakir et al. have managed to image thousands of RBCs in a single FOV and train a DL model to determine the infection status of the quantitative phase image of a segmented cell.^22^ Their system achieved a theoretical resolution of 0.86 μm, FOV of 7540 × 6340 pixels, and took approximately 20 min to collect a stack of images. Their work accurately identified infected cells at a rate of 91% specificity and 72% sensitivity for the machine learning and 98% specificity and 57% sensitivity for the DL model. The results highlight the potential for AI using quantitative phase imaging for digital pathology. To improve the efficiency of their method, multiplexing could be used to significantly reduce the acquisition rate without hindering the accuracy. A minor drawback is that the algorithm had errors in false positives and negative diagnoses of infected RBCs.

**FIGURE 10:**
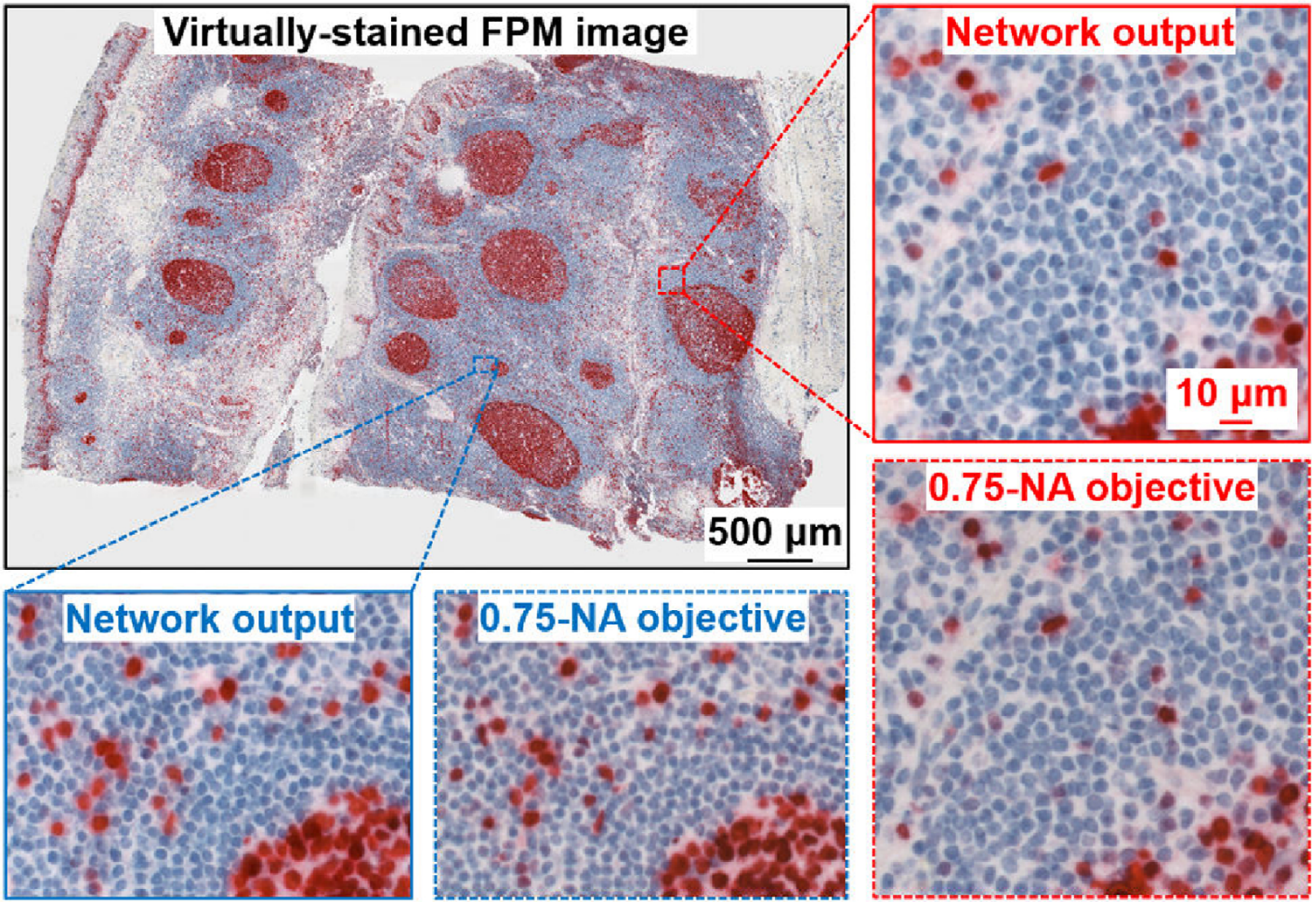
Immunohistochemistry slide, which is virtually stained using DL and captured through FPM. The acquisition time was ≃ 2 min with a maximum synthetic NA of 0.55 (figure originally presented in Wang et al.^21^).

As previously mentioned, colour FPM has been used for potential digital pathology applications and is limited by coherent artefacts, which do not exist in incoherent images.^3^, ^15^ To combat this, Wang et al. have used DL‐FPM for noninvasive virtual fluorescence staining on mouse kidneys (shown in Figure 10).^21^ They trained a cycle‐consistent adversarial network with multiscale structure similarity loss to execute virtual brightfield and fluorescence staining of images recovered through FPM. Their approach managed to reduce the coherent artefacts and create a similar colour image to one captured with a control microscope. However, colour FPM is time‐consuming, and the process becomes even longer with the addition of a neural network. Therefore, these factors are currently preventing its use in clinical digital pathology.

UV Fourier ptychography is a promising avenue for biological research. It has been used for label‐free biochemical imaging, nanoscale imaging of semiconductor devices (achieving a resolution below 100 nm).^24^, ^97^ It is rapidly advancing, with several papers published last year focusing on optimising and improving the method.^98^ Deep ultraviolet microscopy is a powerful tool for high‐contrast imaging of cellular structures.^90^ It allows label‐free, noninvasive, live‐cell imaging while reducing the photodamage compared to other UV imaging methods.^99^ Biomolecules at UV wavelengths have an intrinsic absorbance, which leads to autofluorescence (which can lead to issues with specificity). Biological cells contain aromatic amino acids, nucleotide bases, and drug molecules, which absorb UV light. The adaptation for deep UV‐FPM is a different LED array, which emits light within the UV range. Zhao et al. demonstrated digital pathology applications of deep UV‐FPP by analysing label‐free chemical imaging of unstained biological specimens: HeLa cells, blood smears and skin tissue sections.^99^ Their work in all three specimens highlighted the superior contrast achieved through their method compared to a standard microscope (at least 10× the amount, yielding a resolution of 345 nm). Consequently, they resolved nuclear features that could not be observed using a typical microscope. They proposed that their method could be used in chromatin organisation and nuclear dynamics to improve intraoperative consultation by reducing the overall time for analysis.^99^ They theorised that further developments of their technique could achieve higher resolution, and incorporating neural networks would advance digital pathology. One necessary improvement would be to increase the processing speed, as the algorithm uses significant computational time, reducing its effectiveness in digital pathology.^9^ By reducing the computational time, the technique would be invaluable for clinical pathology, for example, consultation time would be dramatically improved compared with the lengthy process of staining procedures.

### 3.6 Infrared FPM

One significant disadvantage of FPM was its ability to accurately model the light interaction with thick samples.^80^ Other methods, such as ODT, have been used in combination with FPM to allow thick sample imaging (Section 3.3.2). Another example method is hemispherical digital condensers (HDCs), which are arrays of LEDs arranged to allow an ideal constant LED distance to the sample, providing better illumination uniformity when compared with planar LED arrays. Sen et al. were the first to apply HDCs to Fourier ptychography, which allowed plane waves from varying angles at a higher NA than most standard FPM setups.^100^ Their setup used near‐infrared light to image photonic crystals and showed that for samples with a uniform period and direction, the images are limited by the Rayleigh criterion (the minimum distance to resolve point sources), not the Abbe diffraction limit.^101^

Since then, HDCs and near‐infrared light have been applied to FPM in several medical and research applications. For example, Pan et al. have used HDCs to achieve an effective imaging performance by equipping an elaborate 3D printed HDC with adjustable brightness and 0.95NA, highlighting the potential use in digital pathology, infrared, and noninvasive 3D live cell imaging.^4^, ^102^ A practical example of HDCs in an FPM setup is for high‐resolution 3D imaging of in vitro live samples.^53^ To do this, a thick sample is modelled as a superposition of multiple thin slices. As the light propagates through each slice and scatters into a complex field, it is thought of as the incident light of the next slice. The 3D spectrum transmission is modelled using the Rytov approximation in combination with the aberration recovery algorithm to allow the reconstruction of a 3D high‐resolution scattering potential.^2^ Multiplex coded illumination was used to reduce the acquisition time further, enabling fast acquisition 3D imaging at high resolution. Their method was successfully shown to image a USAF target and COS‐7 cells and has clear potential for other digital pathology applications. However, minor miscalibrations can affect the quality of the reconstruction, and therefore, its ability to be used in clinical digital pathology is still limited.

## 4 ADVANCED ALGORITHMS AND THEIR POTENTIAL USE IN DIGITAL PATHOLOGY

With the rapid expansion of FPM as a field of computational imaging, more intricate algorithms and AI techniques are being developed. This section details three computational methods that will likely profoundly impact the field of FPM, specifically on improving digital pathology.

### 4.1 AA‐P algorithm

**FIGURE 11:**
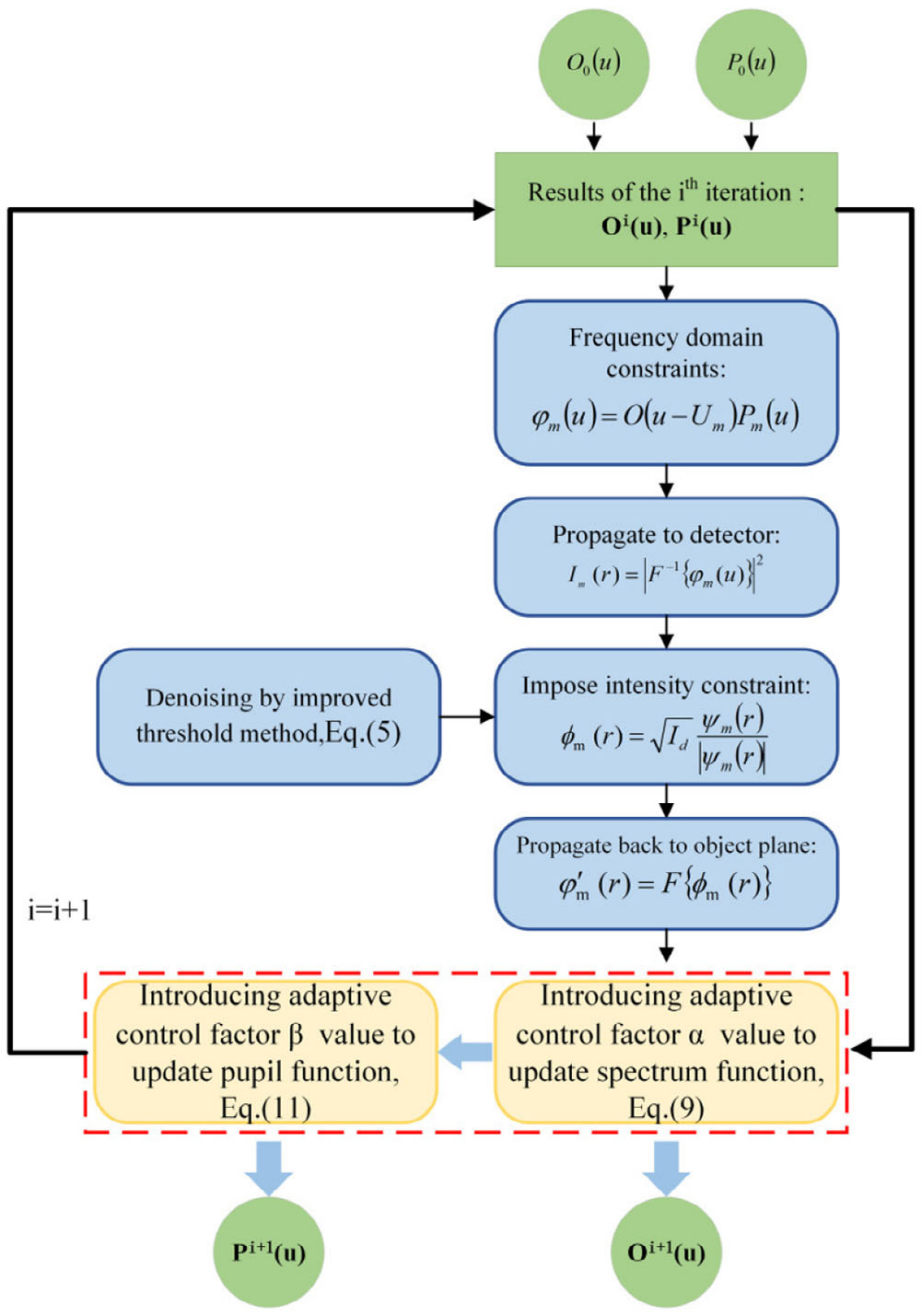
Flowchart of the AA‐P algorithm.^18^ $O_{0}\left(u\right)$ is the initial high‐resolution spectrum and $P_{0}\left(u\right)$ is the light pupil function. The pupil function and high‐resolution spectrum are progressively updated through iterations of the algorithm, shown by $O^{i}\left(u\right)$ and $P^{i}\left(u\right)$. After determining the frequency‐domain analysis, a constraint is imposed on the intensity after propagation to the detector. The result is projected back to the object plane, before adaptive control factors are added to the pupil and spectrum functions, and the process is repeated (figure used with credit to Wu et al.^18^).

Ruofei et al. proposed an FPM aberration correction reconstruction algorithm (AA‐P) based on an improved phase recovery strategy alongside machine learning (Figure 11 shows a flowchart of the algorithm).^18^ The AA‐P algorithm has a faster rate of convergence and is highly robust compared to other standard FPM algorithms.^28^ The AA‐P optimises the updating process of the optical pupil and sample spectral functions by reacting and selecting a modulation factor from the acquired dataset; it iteratively estimates the missing phase by alternating between the image and frequency domains.^18^ The algorithm then alternates projecting the estimate into the image space and the current estimate into the Fourier domain. The image space is adjusted based on prior information about the object, and in the Fourier domain, the reconstructed image needs to match the data in amplitude and phase. Therefore, using a CNN, the algorithm can actively adapt to aberrations within the optical system.

The algorithm was verified by analysing open‐source biological samples, highlighting its potential use in digital pathology. Compared to the EPRY algorithm, the reconstruction quality was shown to be 82.6% more accurate while having a faster run time of 17 s, compared to 20 s. However, the AA‐P focuses on overall aberration correction and has limitations for individual‐focused items. Liu et al. proposed an adaptation that accurately predicts defocus distances for different biological samples.^28^ In their work, they analysed the effectiveness of the AA‐P algorithm in predicting defocus distances across different biological samples such as HE pathological tissue, human blood smears and mouse kidneys. They found that the AA‐P algorithm had better reconstruction quality, higher aberration robustness, and better convergence performance. They predict that the AA‐P algorithm could be used for dynamic aberration correction when observing live samples, allowing high‐throughput quantitative phase imaging. However, to get to this stage, more work focused on improving the imaging efficiency of FPM is required.^18^

### 4.2 Multilook FPM

Due to its sensitivity to alignment, FPM is currently limited in clinical practices, as LED array drifting or liquid droplets within the system can drastically harm the phase‐retrieval process. In addition, the process for realignment is highly complex; therefore, once misaligned, the setup requires an expert to be fixed. However, Bianco et al. have proposed a more robust FPM setup resistant to misalignment.^103^, ^104^ The setup is capable of whole slide imaging (achieving a FOV of 3.3 ${\mathrm{mm}}^{2}$ and a resolution of 0.5 μm), using FPM and incorporates DL to correct alignment errors such as drifting of the LED array. They propose a blind method, Multi‐Look FPM (ML‐FPM), which consistently recovers the object complex amplitude of the whole FOV and converges to the correct quantitative phase contrast of the specimen without preestimation. It has been used to monitor fibroblast cellular behaviour on particular micropatterned substrates.^105^ They demonstrated that FPM can be used as an effective imaging tool for multiscale monitoring of cell distribution. However, ML‐FPM currently has drawbacks that make it (presently) unusable for diagnostic purposes. Due to its high computational demand, an average computer will take a significant amount of time to run the neural network, and this problem becomes significantly worse when trying to image live cells or any video attempts. Another issue is that the deep neural network needs to be trained sufficiently and correctly by the user; if trained incorrectly, it will potentially be unable to generate the correct high‐resolution images and even add artefacts. With advancements in computing and further training, there is still potential for ML‐FPM to be used to help digital pathology.

### 4.3 Alternating direction of multipliers method (ADMM)

The alternating direction method of multipliers (ADMM) is a powerful algorithm well suited to distributed convex optimisation. The algorithm uses decomposition‐coordination principles in that the solutions to small localised problems are coordinated to find an overall solution to a large global problem in the system.^106^ Chang et al. proposed a generalised version of the algorithm, which guarantees convergence.^19^

**FIGURE 12:**
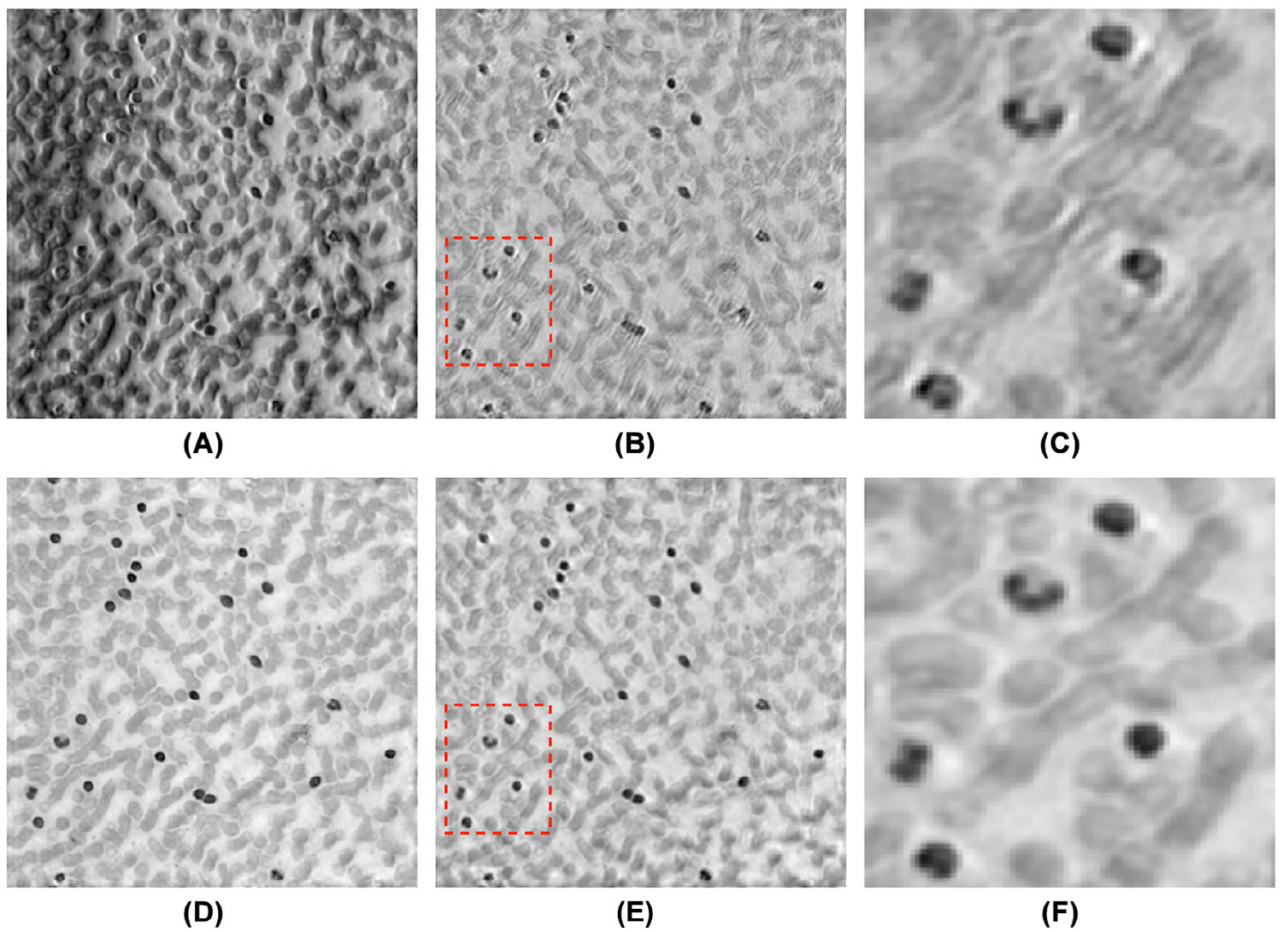
Experimental datasets used to exhibit the various reconstruction results of different algorithms. (A–C) The EPRY's reconstructed amplitude images. (D–F) The BADMM's reconstructed images. C and F are the ROIs in B and E, respectively, which show the clear improvement by BADMM. A, D and B, E are the reconstructed results of the dataset where one of every three and one of every six images were selected, respectively (figure taken from Yang et al.^107^).

Yang et al. have proposed a batch‐based modification of the ADMM.^107^ The key difference in the algorithm is that it updates variables using only part of the measurement data per loop, compared to using all of the data to complete updates to the variables. Their results highlighted that the algorithm converges faster (taking approximately 500 s for 230 epochs) and more robustly than the EPRY. In addition, upon comparison with the EPRY algorithm, processed images of the same sample site were clearer. They had higher resolution, highlighting their superiority (a sample of the results from their work is shown in Figure 12).

More recently, the algorithm has been combined with untrained generative networks to solve Fourier phase retrieval problems.^108^ By modelling the problem as a constrained optimisation problem and then modifying the ADMM algorithm to solve it. Termed the Net‐ADM, it has theoretically been proven to have a solution to the established model. The algorithm showed higher accuracy than other modern algorithms and had stronger robustness when exposed to Gaussian noise than other algorithms.

### 4.4 FPM Whole slide imaging (WSI) algorithms

**FIGURE 13:**
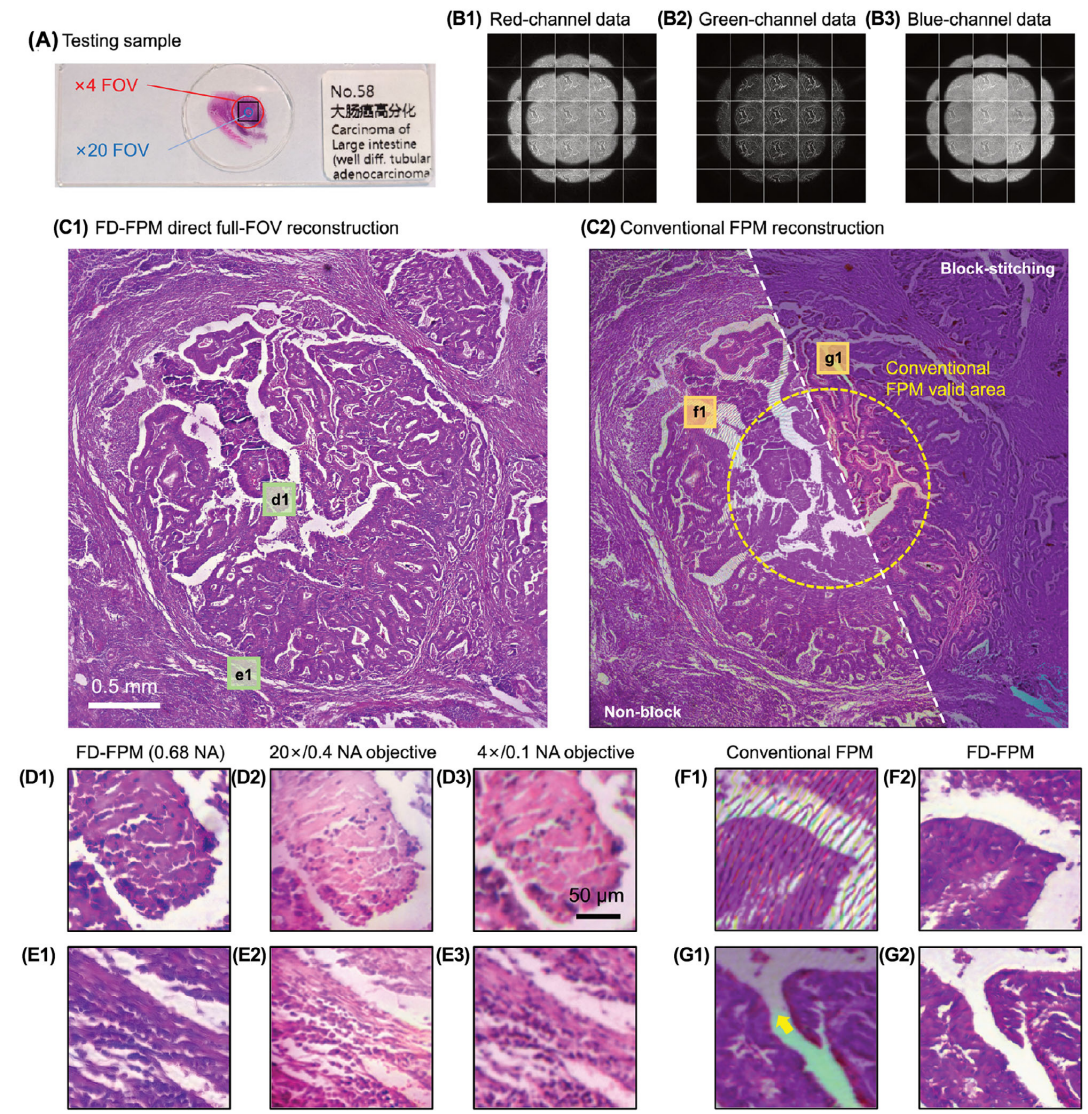
Full‐colour FPM constructions for a pathology slide. (A) The testing sample (human colorectal carcinoma section). (B1–B3) Raw data of three colour channels, which exhibit the common vignetting effect. (C1) Direct full‐FOV reconstruction using their technique (FD‐FPM). (C2) The full‐FOV reconstruction using conventional FPM (EPRY). (D1, E1) Magnified images of two RIOs in (C1). (D2, E2) and (D3, E3) are corresponding images captured by a colour sensor with a 20× and 4× objective lens, respectively, for comparison. (F1) ROI in yellow dotted line, which shows the severe vignetting artefacts in non‐blocked reconstruction of EPRY. (G1) ROI with colour difference and stitching artefact in reconstruction of EPRY. (F2, G2) ROI magnified images of FD‐FPM reconstruction corresponding to F1, G1. Figure taken from Zhang et al.^109^

Block reconstruction and stitching are often necessary procedures in FPM to reduce the number of vignetting artefacts. However, as mentioned, these standard techniques introduce digital artefacts through unavoidable uncertainty in algorithms or DL models. Recently, Zhang et al. have developed a novel method using feature‐domain FPM (FD‐FPM) inspired by the structure‐aware forward model to achieve stitching‐free, full‐FOV reconstruction.^109^ Their work obtained a synthetic NA of 0.68 (compared to the objective NA of 0.1) while maintaining a large FOV of 3.3 × 3.3 ${\mathrm{mm}}^{2}$, and completely removing artefacts found upon comparison with standard EPRY algorithms (see Figure 13 for a full pathological slide image). Their method proposes a structure‐aware forward model using a trained network to detect image structures. The image's gamma is first adjusted to enhance the darkfield images, before extracting first‐order edges as their feature set. They then minimise the sum of absolute differences between the predicted and measured edges. As vignetting is a low‐frequency brightness fall‐off, it becomes suppressed when comparing only sharp edge maps of the image.^110^, ^111^ The algorithm was used on standard (cameraman) and full‐colour FPM datasets and has several additional benefits to improve pupil function recovery. One of these is local aberration recovery; by dividing the full‐FOV raw data into small segments, reconstructing and stitching them, they can assign a specific aberration‐correction pupil function to each region. Another advancement in their algorithm is computational refocusing; high magnification objectives used in WSI systems often struggle to precisely focus images due to the thickness of the sample and their 3D nature. However, the algorithm was able to successfully refocus at each stage of the imaging process.

Another example of WSI achieved due to neural algorithms is FPM with implicit neural representations (FPM‐INR).^112^ In early methods of DL‐FPM, a z‐stack of images is generated using the same architecture as the physical z‐stack. This process takes a long time to reconstruct due to the storage of the high‐resolution, volumetric set. Zhou et al. have developed a novel, more efficient DL method where they encapsulate the physical *z*‐stack data into a compact feature volume coupled with the weights of a small neural network.^112^ To do this, they built a continuous neural representation of the sample, which factorised the volumetric features. In the neural representation, two multilayer perceptrons map each feature vector to a real‐valued amplitude and phase, respectively, to reconstruct the complex field. The model is then trained on alternating defocus sampling (changing between predefined depths and random depths, via selecting z values for optimisation at different epochs) to efficiently reconstruct the 3D image stack. Their work successfully removed artefacts in (30 μm sample‐thickness) thyroid gland images compared to standard FPM and has clear potential application in digital pathology.

As mentioned, modelling light‐sample interaction can be a problem in FPM when using thick cytological smears, and other ptychography implementations can work more efficiently. Jiang et al. have developed a handheld, multiplexed, and AI‐powered ptychography whole‐slide scanner.^113^ Their work no longer needs to be limited by the thickness of the sample.

The scanner can resolve 388 nm, acquiring gigapixel images with a 14 mm × 11 mm area in approximately 70 s, which was validated through imaging of a resolution target, pathology slides and malaria‐infected blood smears. The device can recover the quantitative phase information, providing a tool for visualising the cellular topographic structure in 3D. This example highlights one of the key downsides of FPM, although improvements to 3D imaging have been made, typical FPM methods still struggle to image thick samples >10 μm.

Overall, several advanced algorithms have been developed to advance FPM and its use in digital pathology. A summary is outlined in Table 1, highlighting the algorithm, its area of use in FPM and the benefits and disadvantages of each. These more robust algorithms and techniques, combined with FPM, allow higher‐resolution and more accurate images essential for digital pathology. In particular, WSI methods reduce or completely remove artefacts while improving the acquisition rate, and maintaining the high resolution and field of view. Therefore, WSI techniques will likely be strong candidates in the advancement of digital pathology utilising FPM.

**TABLE 1:** Details of the various algorithms outlined in the paper, listing their areas of use, and the benefits and disadvantages of each.

Algorithm	Area of use in FPM digital pathology	Benefits	Disadvantages
Gerchberg–Saxton^10^, ^12^, ^13^	Generally used in most early FPM methods.	Gerchberg–Saxton is (1) relatively straightforward to implement and (2) requires low computational demands.	However, it has (1) slow convergence, (2) low image quality compared to modern methods, (3) tendency to struggle with large datasets.
EPRY^8^, ^26^, ^31^, ^56^	Fundamental advancement which is used in most areas of FPM.	The EPRY algorithm (1) allows recovery of the Fourier spectrum and pupil function simultaneously; (2) drastically improving image quality; (3) significantly reduces acquisition time by removing the need for pupil characterisation data; (4) handles alignment drift better.	Although EPRY was a significant advancement, it still has two downsides compared to modern algorithms: (1) It is sensitive to noise and therefore, needs to be used in combination with other algorithms; (2) It is slow for high‐throughput data.
Wirtinger–Flow^39^, ^40^	Commonly used in combination with other modern methods.	Wirtinger–Flow (1) reduces exposure time for Gerchberg–Saxton by 80% and (2) handles noise well; (3) is robust and drastically improves convergence.	Wirtinger‐Flow has: (1) a longer running time; (2) vulnerability to speckle noise and alignment errors; (3) struggles regarding WSI for scalability.
AA‐P^18^, ^28^, ^107^	Pathology methods which are susceptible to high aberrations such as H&E stained pathological tissue, and blood smears. There is potential for live‐cell imaging.	The AA‐P algorithm has several benefits: (1) adaptive correction of aberrations throughout the image acquisition process; (2) improves iterative image quality; (3) short run time approximately 17 s; (4) low image distortion and crosstalk; (5) excellent for high‐throughput data.	However, there are disadvantages: (1) it is limited to individual corrections; (2) phase reconstruction results have lower contrast; (3) There is still blurring compared to more advanced methods; (4) it requires a large dataset and good computer memory.
Multi‐Look^103^, ^104^, ^105^	Any imaging sensitive to misalignment, i.e., multicell monitoring, whole slide imaging. Exceptionally useful in imaging with scattering media and low‐signal samples.	Multilook allows (1) highly robust correction to misalignment, while actively correcting it; (2) improving image quality; (3) removing phase errors.	Yet, Multi‐Look suffers from (1) a long processing time; (2) powerful GPU requirements; (3) needing a large dataset.
ADMM^19^, ^106^, ^107^	Imaging methods with substantial noise, i.e., darkfield imaging, blood cell imaging where noise tends to collect around the cellular walls. Can be used in whole slide imaging.	ADMM is exceptional at (1) reducing the effects of noise interference in images and (2) phase and darkfield information recovery, and therefore improving the image quality.	Due to it being great at analysing noise, it needs to be computationally intensive, requiring significant data and powerful software.
Neural networks and DL^28^, ^37^, ^63^, ^64^, ^92^, ^93^, ^94^, ^95^, ^96^	Whole slide imaging, thick tissue and other imaging.	Neural networks and DL have many benefits: (1) they remove artefacts from the images; (2) improve image quality, (3) allow local aberration recovery; (4) capable of computational refocusing; (5) once trained, allow a fast acquisition time; (6) significantly reduce the memory usage due to better optimisation of data; (7) allows thick sample imaging; (8) leading potential for end‐to‐end pipelines.	Neural networks and DL will likely be the future of FPM and digital pathology, however: (1) they need to be coded specifically for the task, and therefore, training is reliant on the user's expertise; (2) training the network can take a significant amount of time (up to days or even weeks); (3) they require powerful computers and large datasets.
Vectorial pFPM^42^, ^56^, ^57^, ^62^, ^63^, ^64^, ^65^	Polarised Fourier ptychography imaging, built upon the Gauss–Newton method used in Fourier ptychography topography.	Vectorial pFPM is capable of (1) highly accurate phase retrieval; (2) reducing computational intensity; (3) exceptionally reducing the noise in birefringent imaging systems; and (4) fast total reconstruction time (approximately 200 s for 300 μm × 300 μm FOV).	However, it is: (1) specific to pFPM; (2) limited to thin samples; (3) needing improvement to account for partial coherence of LED illumination; (4) computationally demanding.

## 5 DISCUSSION

Fourier ptychography microscopy is an accurate, high‐resolution, large FOV technique capable of high‐throughput and high‐resolution imaging, making it an ideal tool for digital pathology. Several applications of FPM have focused on improving digital pathology through various observation methods: UV fluorescence, label‐free imaging, visible light, infrared and polarisation imaging, successfully showing its use in imaging various biological specimens and high potential in digital pathology.

Physical methods have focused on adapting the FPM setup to improve the resolution and FOV, allowing better imaging for digital pathology. Multiplexed coded illumination is the most impactful and will likely be adapted to all FPM setups in the future, as although computationally complex, it drastically reduces the acquisition time while maintaining the high‐resolution and large FOV. A practical modification to the FPM setup is replacing the planar LED array with a more advanced dome‐shaped array, which significantly reduces the required number of LEDs and creates steeper angles that capture more information and have better overlap, improving the SNR. However, calibration issues currently limit their ability to be used in clinical digital pathology. Hemispherical digital condensers can be a promising adaptation as they create better angles, increasing the NA.^100^ It has been used in advanced 3D digital pathology live cell analysis.^53^, ^102^ However, like dome arrays, calibration problems can negatively impact the process (although not as significant an issue).

Phase‐retrieval algorithms or DL approaches are specific to the area of FPM's requirements. The first algorithm adapted specifically for FPM was the Gerchberg–Saxton, which has been modernised for use in digital pathology. Rapid colour FPM and pFPM use the Gerchberg–Saxton algorithm for high‐throughput digital pathology, where the error is marginally larger (>1.5%) than other FPM methods. Yet, the number of artefacts in the image is reduced. Colour‐transfer FPM methods are also notably faster than conventional FPM techniques, with a processing time of less than 1 s. It is particularly useful when recovering from a single wavelength because the chromatic aberration effects are less than those of other colour FPM methods. Modern digital pathology FPM uses various algorithms explicitly tailored to the imaging problem. The EPRY algorithm is commonly used in pFPM, as it measures the real pupil function phases and iteratively updates them, allowing it to continuously correct aberration problems within the system. This technique has been used to analyse and diagnose liver tissue for fibrosis and, in the future, will be an invaluable tool in preventing liver cirrhosis. Another valuable use of this technique will be its ability to correct birefringence problems in plastic lenses, improving low‐cost microscopes' ability to analyse anisotropic biological samples.

DL is a critical approach used to improve algorithms in FPM, improve image reconstruction, and allow automated diagnosis in digital pathology. DL has been used to actively correct phase retrieval functions within algorithms, increasing their accuracy and allowing them to be applied to all areas of FPM. In addition, it is currently being combined with fluorescence Fourier ptychography to automate the detection of malaria parasite infections, label‐free detection of HeLa cells, and resolve nuclear features within other biological cells. It has clear potential for digital pathology in the future. Deep neural network training is required for new fields and progression in the current uses of digital pathology.

More advanced computational techniques like the AA‐P, ML‐FPM, and ADMM are critical in meeting high‐throughput digital requirements. They are more accurate than the Gerchberg–Saxton, Gauss–Newton and EPRY algorithms while better coping with noisy environments. Therefore, their use will likely be prevalent in digital pathology imaging in the future, where high throughput is the main priority. Table 1 summarises the algorithms and/or other computational methods.

In systems with significant aberration problems, the AA‐P algorithm will be especially useful in predicting defocus distances in several biological samples. Finally, WSI techniques are the most advanced when removing artefacts commonly generated due to vignetting, while maintaining a large FOV and high resolution. Therefore, WSI methods appear to be an essential technique for digital pathology in the future.

CNNs have been used to continuously correct and improve images during reconstruction. It has clear potential in digital pathology as it expands the imaging depth‐of‐field and provides a potential way of solving the digital refocusing problem using DL. Currently, FPM is used more as a proof‐of‐concept area of research rather than being built for practical clinical use, as it is still in its infancy. However, by designing algorithms that account for more practical errors, such as drift, FPM will continue to get closer to being used in clinical digital pathology. The balance between precision in reconstruction algorithms and time constraints when needing high throughput is still problematic. However, with advancements in computing, unique algorithms have built upon the fundamental concepts and used them to advance FPM. They have shown the benefit of FPM in whole slide imaging, virtual staining, fluorescence and other digital pathology applications. Fourier ptychography has been used in different biomedical applications in clinical and research fields, such as drug screening and X‐ray imaging.^19^, ^23^, ^114^ Improvements in these external fields will also likely lead to developments in FPM for digital pathology.

## 6 CONCLUSIONS

FPM can create a large FOV, high‐resolution image ideal for digital pathology. With improvements to algorithms unique to the pathology requirements, FPM will be a critical tool in developing automatic diagnosis. With the rapid advancement of AI, DL, and improvements to phase‐retrieval algorithms, FPM will be a precise tool of the future for accurate, high‐throughput digital pathology across all fields of medical research and clinical work. In addition, physical improvements to all setups, such as the dome array and nonphysical enhancements, such as multiplexed coded illumination, will also help achieve accurate digital pathology. There is clear potential for automated digital pathology, but for clinical use, the tradeoff between precision and acquisition time needs to be improved to become accurate enough while ensuring the method remains practical.

## CONFLICT OF INTEREST STATEMENT

The authors declare no potential conflict of interest.
